# Supplementary material for: A novel reaction-diffusion architecture for engineering self-organized patterns in mammalian cells
Source: bioRxiv. 2026 May 25:2026.05.24.727552. Preprint. [Version 1] doi: 10.64898/2026.05.24.727552 (PMC13232235; doi:10.64898/2026.05.24.727552)
Supplement: Supplement 5 [file NIHPP2026.05.24.727552v1-supplement-5.pdf]

bioRxiv preprint doi: https://doi.org/10.64898/2026.05.24.727552; this version posted May 25, 2026. The copyright holder for this preprint (which was not certified by peer review) is the author/funder, who has granted bioRxiv a license to display the preprint in perpetuity. It is made available under a CC-BY 4.0 International license.

Description of Supplementary Materials Attached to this Preprint

Supplementary Movie 1: Timelapse microscopy of L929 mouse fibroblasts engineered with a synthetic reaction-diffusion circuit, composed of synNotch-mediated juxtacrine activation and paracrine inhibition (JAPI), showing the activation of the ligand (GFP) over time.

Supplementary Movie 2: Two-dimensional simulation of a juxtacrine activator–paracrine inhibitor (JAPI) reaction–diffusion circuit, parameterized to recapitulate the phenotype of an equivalent synthetic circuit implemented in mammalian fibroblasts using synNotch-based components.

Supplementary Movie 3: Two-dimensional simulation of a juxtacrine activator–paracrine inhibitor (JAPI) reaction–diffusion circuit, parameterized to recapitulate the phenotype of an equivalent synthetic circuit implemented in mammalian fibroblasts using synNotch-based components. Here, the simulation domain size is reduced (20 × 20), mimicking experimental conditions in which fibroblasts carrying the JAPI circuit are aggregated into small spheroids generated from 500 initial cells.

Supplementary Movie 4: Time-lapse imaging of L929 mouse fibroblasts engineered with two orthogonal synthetic reaction–diffusion circuits, each composed of synNotch-mediated juxtacrine activation coupled to paracrine inhibition (JAPI). The video shows the spatiotemporal evolution of the expression of the two membrane-tethered activators, GFP and mCherry, over four days.

Table 1. Parameter sets used to generate the 1D and 2D simulation outcomes shown in the main and supplementary figures for single-circuit reaction–diffusion systems implementing either juxtacrine activator–paracrine inhibitor (JAPI) or paracrine activator–paracrine inhibitor (PAPI) architectures. The table specifies initial conditions, activator type, simulation dimensionality (1D or 2D), step number limit, and all simulation parameter values.

bioRxiv preprint doi: https://doi.org/10.64898/2026.05.24.727552; this version posted May 25, 2026. The copyright holder for this preprint (which was not certified by peer review) is the author/funder, who has granted bioRxiv a license to display the preprint in perpetuity. It is made available under a CC-BY 4.0 International license.

Table 2. Parameter sets used for 2D simulations of dual interacting reaction–diffusion circuits. For each circuit, the table lists the full parameter set of both circuits, together with additional variables specific to the dual-circuit architecture, including co-initiation conditions and cross-inhibition parameters.

Table 3. Engineered cell lines generated and used in this study, including where they appear in the figures, a description of the circuit, and the lentiviral vectors used for assembling the circuit. Vector descriptions and corresponding Addgene accession numbers, where available, are provided.

Table 4. Dual-input promoters engineered in this study, including information on the downstream the target transgene, the number and arrangement of binding sites for each of the two transcriptional inputs, the associated barcode for recognition in a pooled library assay, and the full promoter sequence.

bioRxiv preprint doi: https://doi.org/10.64898/2026.05.24.727552; this version posted May 25, 2026. The copyright holder for this preprint (which was not certified by peer review) is the author/funder, who has granted bioRxiv a license to display the preprint in perpetuity. It is made available under a CC-BY 4.0 International license.

Supplementary Figures

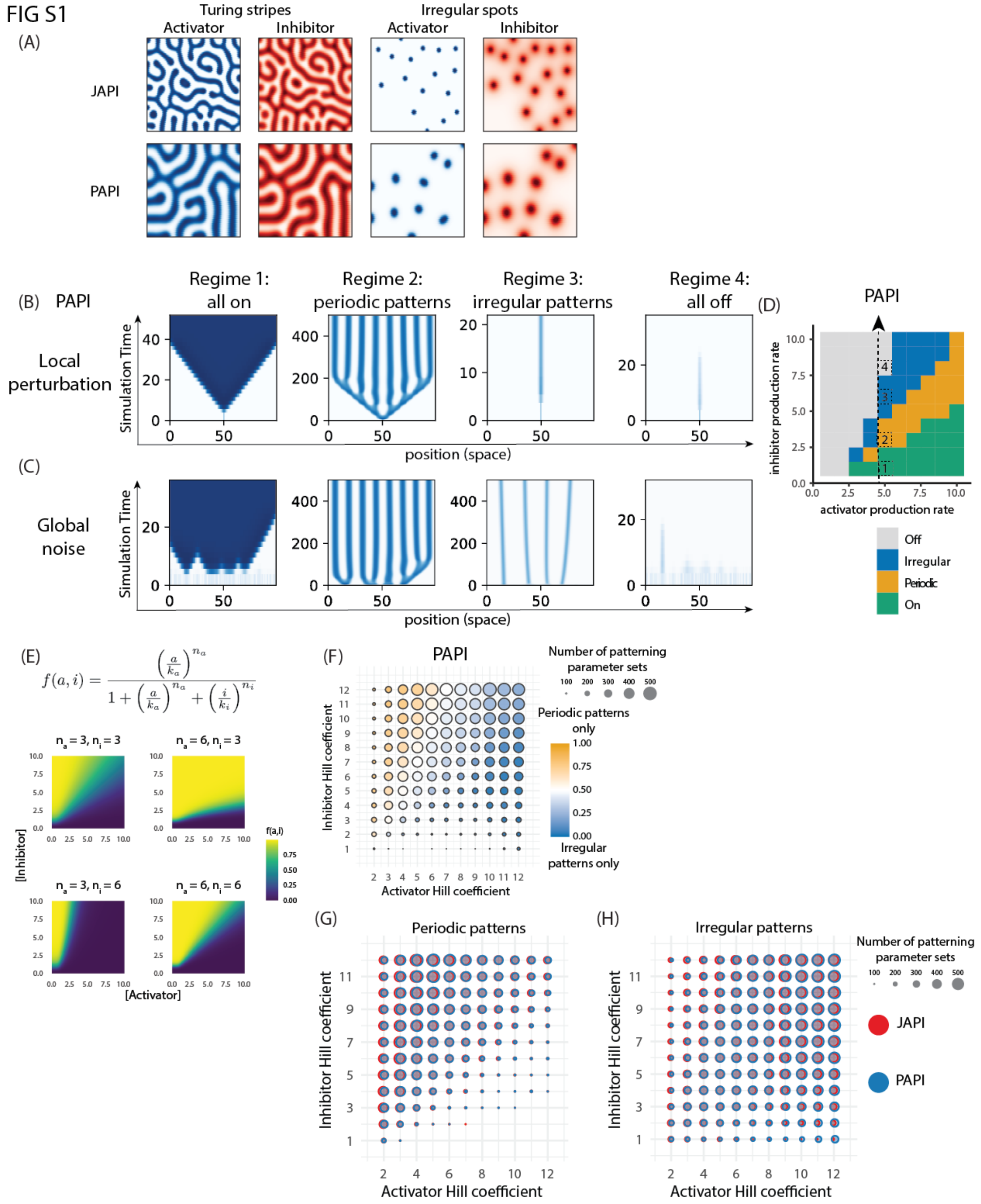

Fig. S1, related to Fig. 1. JAPI and PAPI architectures access the same patterning regimes.

bioRxiv preprint doi: https://doi.org/10.64898/2026.05.24.727552; this version posted May 25, 2026. The copyright holder for this preprint (which was not certified by peer review) is the author/funder, who has granted bioRxiv a license to display the preprint in perpetuity. It is made available under a CC-BY 4.0 International license.

(**A**) Endpoint snapshots of two-dimensional simulations for JAPI (top row) and PAPI (bottom row) circuits, shown separately for the activator (blue) and inhibitor (red) channels, in two non-homogeneous regimes labeled above each pair of columns (Turing stripes, irregular spots). Intensity indicates dimensionless activator or inhibitor concentration. Simulation setup and parameter values per each simulation is in Methods, Numerical Simulations. (**B-C**) Kymographs of one-dimensional PAPI simulations from a centrally located activator burst (B) or from spatially uniform noise (C), for parameter sets representative of the four regimes, columns labeled at the top. Blue intensity indicates dimensionless activator concentration (see Supp. Note 3 for description of dimensionless units). Initial conditions and parameter values per each simulation is in Methods, Numerical Simulations. (**D**) Phase diagram of regime classification for a PAPI circuit as a function of activator production rate (x axis) and inhibitor production rate (y axis), with the remaining dimensionless parameters held constant ($n_a = n_i = 3$, $\gamma = 0.5$, $D = 10$). Tiles are colored by regime as indicated in the legend on the figure. The four dashed boxes labeled 1 to 4 mark the parameter values used for the corresponding regimes in (B) and (C). Classification rule in Methods, Defining Patterning Regimes. (**E**) Heatmaps showing the outcome of a competitive-inhibition Hill function shown above, as a function of activator concentration (x axis) and inhibitor concentration (y axis), for four (n_a, n_i) combinations indicated above each heatmap. (**F**) Dotplot of PAPI patterning outcomes from a parameter sweep with 1900 per activator (x axis) and inhibitor (y axis) Hill coefficient parameter combinations, taken by independently varying the activator and inhibitor production rates and inhibitor degradation rate. Compare to Fig.1J where the same graph is shown for the JAPI architecture. Dot size is proportional to the number of parameter combinations producing patterns at each (n_a, n_i) pair; dot color indicates the proportion of those producing periodic patterns (orange) versus irregular patterns (blue). Sweep ranges and classification settings in Methods, Defining Patterning Regimes. (**G-H**) Dotplots of JAPI (red) and PAPI (blue) overlaid for parameter sets producing periodic patterns (G) or irregular patterns (H), projected onto the activator Hill coefficient (x axis) and inhibitor Hill coefficient (y axis). Compare to Fig. 1J and S1F, where the same dotplot is shown for each architecture separately with periodic-vs-irregular composition by color. Dot size is proportional to the number of parameter combinations producing the indicated pattern type in each architecture. The horizontal separation between paired circles was set so that their intersection area is proportional to the observed overlap between JAPI and PAPI parameter sets giving rise to the same regime. Sweep ranges and JAPI/PAPI-specific settings in Methods, Defining Patterning Regimes.

bioRxiv preprint doi: https://doi.org/10.64898/2026.05.24.727552; this version posted May 25, 2026. The copyright holder for this preprint (which was not certified by peer review) is the author/funder, who has granted bioRxiv a license to display the preprint in perpetuity. It is made available under a CC-BY 4.0 International license.

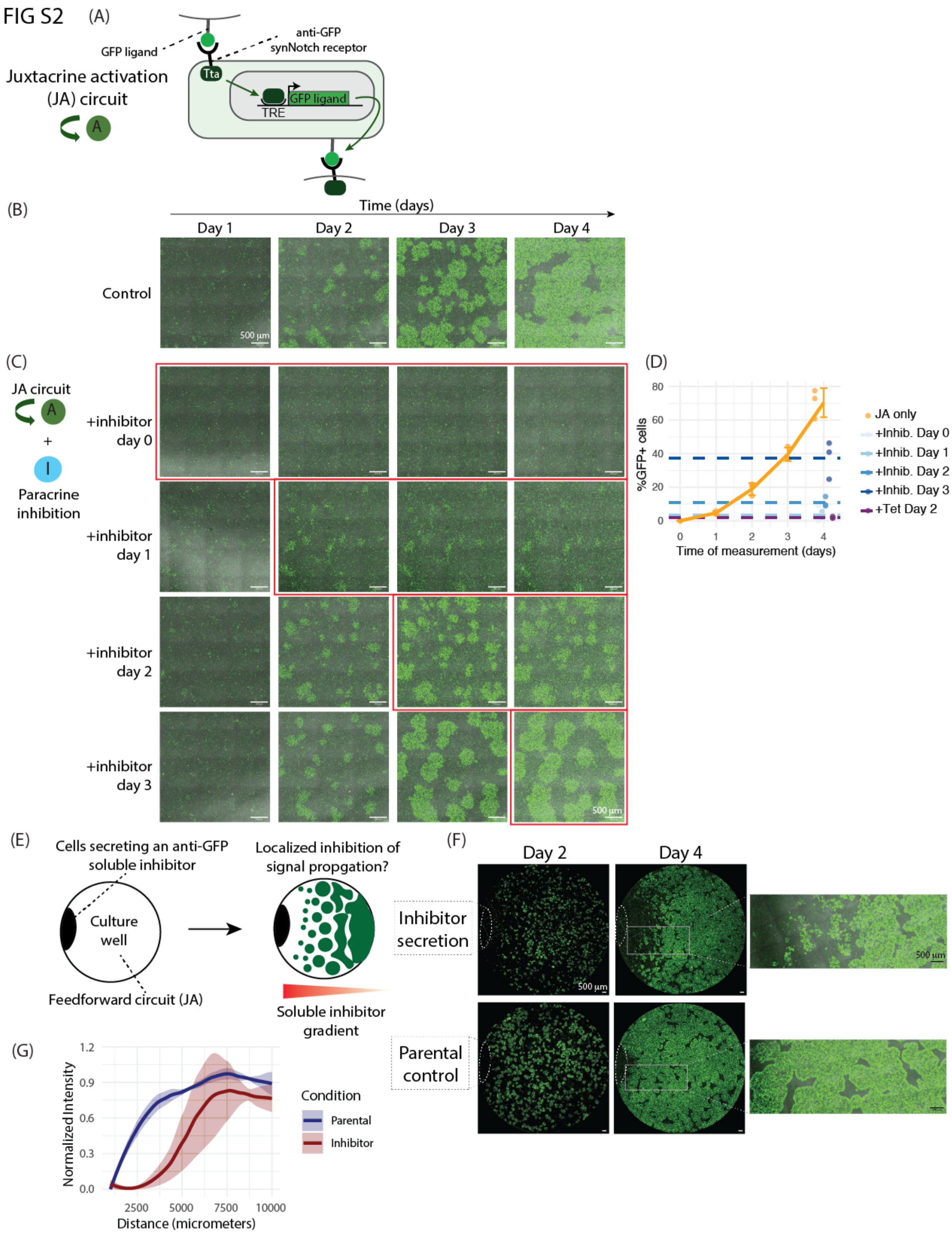

Fig. S2, related to Fig. 2. The juxtacrine activation and paracrine inhibition branches of JAPI are functional and locally acting in isolation.

bioRxiv preprint doi: https://doi.org/10.64898/2026.05.24.727552; this version posted May 25, 2026. The copyright holder for this preprint (which was not certified by peer review) is the author/funder, who has granted bioRxiv a license to display the preprint in perpetuity. It is made available under a CC-BY 4.0 International license.

(**A**) Schematic of the feedforward juxtacrine self-activation (JA) circuit. An anti-GFP synNotch receptor with an intracellular Tta transactivation domain (dark green) detects GFP ligand presented on a neighboring cell, drives transcription from a TRE promoter of a single downstream cassette encoding the membrane-tethered GFP ligand (juxtacrine activator A, green). JA circuit first introduced by Santorelli et al., Nat Comm 2024. (**B**) Fluorescence microscope images at the indicated timepoints of a timelapse experiment, showing the same field of view across time. Cells are L929 fibroblasts containing the JA circuit from (A). Green indicates activated cells (GFP signal); brightfield in grey. Initial condition: homogeneous inactivated cell lawn. Scale bar = 500 µm. (**C**) Fluorescence microscope images at the indicated timepoints of a timelapse experiment of JA cells, showing the same field of view per each row. In the four rows, a soluble anti-GFP inhibitor is added to the culture medium at the day indicated by the red box. Green indicates activated cells (GFP signal); brightfield in grey. Initial condition: homogeneous inactivated cell lawn. Scale bar = 500 µm. (**D**) Line and dot plot of the percentage of GFP-positive cells from experiments as in (C), measured by FACS. The orange line and dots show the uninhibited JA-only propagation timecourse (n = 3). Dots in shades of blue correspond to conditions where the soluble inhibitor was added at the indicated timepoint, all measured at day 4; dashed horizontal lines correspond to the average for each condition (n = 3), drawn across the full axis as a reference level. Cells inhibited with tetracycline, an inhibitor of the Tta intracellular domain of the synNotch receptor, are indicated in purple. (**E**) Schematic of the localized inhibition experimental setup. Left, condition at time zero: cells constitutively secreting soluble anti-GFP inhibitor (black) are locally seeded as a drop at the left edge of the well, and the rest of the well is plated with JA cells (white). Right, cartoon of the predicted outcome after 4 days inside the well: the inhibitor-secreting drop stays on the left edge, a graded distribution of activated GFP cells (green) across the well with fewer activated cells closer to the source. The inferred underlying inhibitor gradient is abstractly depicted as the red triangle at the bottom. (**F**) Fluorescence microscope images at the indicated timepoints of a timelapse experiment of the setup in (E); the same entire well of the experiment is shown at the two time points. Green indicates activated cells (GFP signal); brightfield in grey. Top row, the locally seeded edge cells secrete soluble anti-GFP inhibitor; bottom row, the locally seeded edge cells are non-secreting parental cells. Dotted lines indicate the position of the locally seeded edge cells. Right-most panels show a zoom of the day 4 images at the indicated positions. Scale bars = 500 µm. (**G**) Line plot of normalized GFP intensity as a function of distance from the locally seeded edge cells, for the two conditions in (F). x axis is distance in µm from the seeded cells; y axis is normalized GFP intensity. Curves are means of independent replicates with shading for standard deviation (n = 3). Image processing in Methods, Image Analysis.

bioRxiv preprint doi: https://doi.org/10.64898/2026.05.24.727552; this version posted May 25, 2026. The copyright holder for this preprint (which was not certified by peer review) is the author/funder, who has granted bioRxiv a license to display the preprint in perpetuity. It is made available under a CC-BY 4.0 International license.

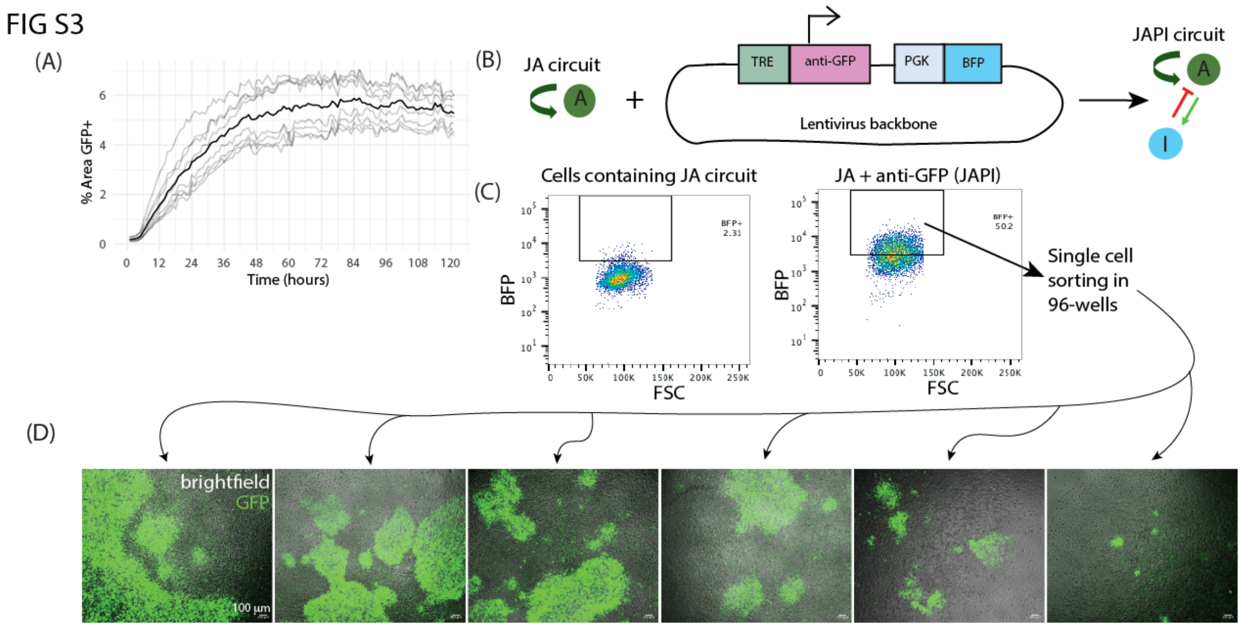

Fig. S3, related to Fig. 2. patterning spontaneously emerges from single sorted JAPI cells. (**A**) Line plot of the area fraction covered by GFP signal over time, measured by live imaging of cells containing the JAPI circuit, from experiments such as shown in Supplemental Movie 1. Light grey curves are individual fields of view (n = 6) captured from two independent experiments; black curve is the average. Initial condition: homogeneous inactivated cell lawn. Image processing in Methods, Image Analysis. (**B**) Schematic of the lentiviral plasmid used to add the paracrine inhibitory module to cells already containing a juxtacrine activation (JA) circuit. The construct encodes the anti-GFP nanobody dimer (paracrine inhibitor) downstream of a TRE promoter, placing inhibitor expression under control of the anti-GFP-Tta synNotch receptor. A constitutive PGK promoter drives expression of a BFP reporter from the same construct, used for sorting in (C). (**C**) FACS plots showing the gating strategy for sorting BFP-positive cells, used to identify cells where the construct from (B) has been successfully integrated. x axis is forward scatter (FSC); y axis is BFP signal. Left, plot read from parental cells containing only the JA circuit (BFP-negative baseline). Right, plot read from cells from the same parental line infected with the construct from (B) (JAPI cells, BFP-positive). The black rectangle marks the gate used to sort BFP-positive cells as single cells into 96-well plates to generate clonal JAPI lines. Percentages indicate the fraction of cells inside the gate. (**D**) Fluorescence microscope images of six independent clonal JAPI cell lines derived from (C), taken directly from 96-well plates used for single-cell sorting, and imaged two weeks after. Green indicates activated cells (GFP signal); brightfield in grey. Scale bar = 100 µm.

bioRxiv preprint doi: https://doi.org/10.64898/2026.05.24.727552; this version posted May 25, 2026. The copyright holder for this preprint (which was not certified by peer review) is the author/funder, who has granted bioRxiv a license to display the preprint in perpetuity. It is made available under a CC-BY 4.0 International license.

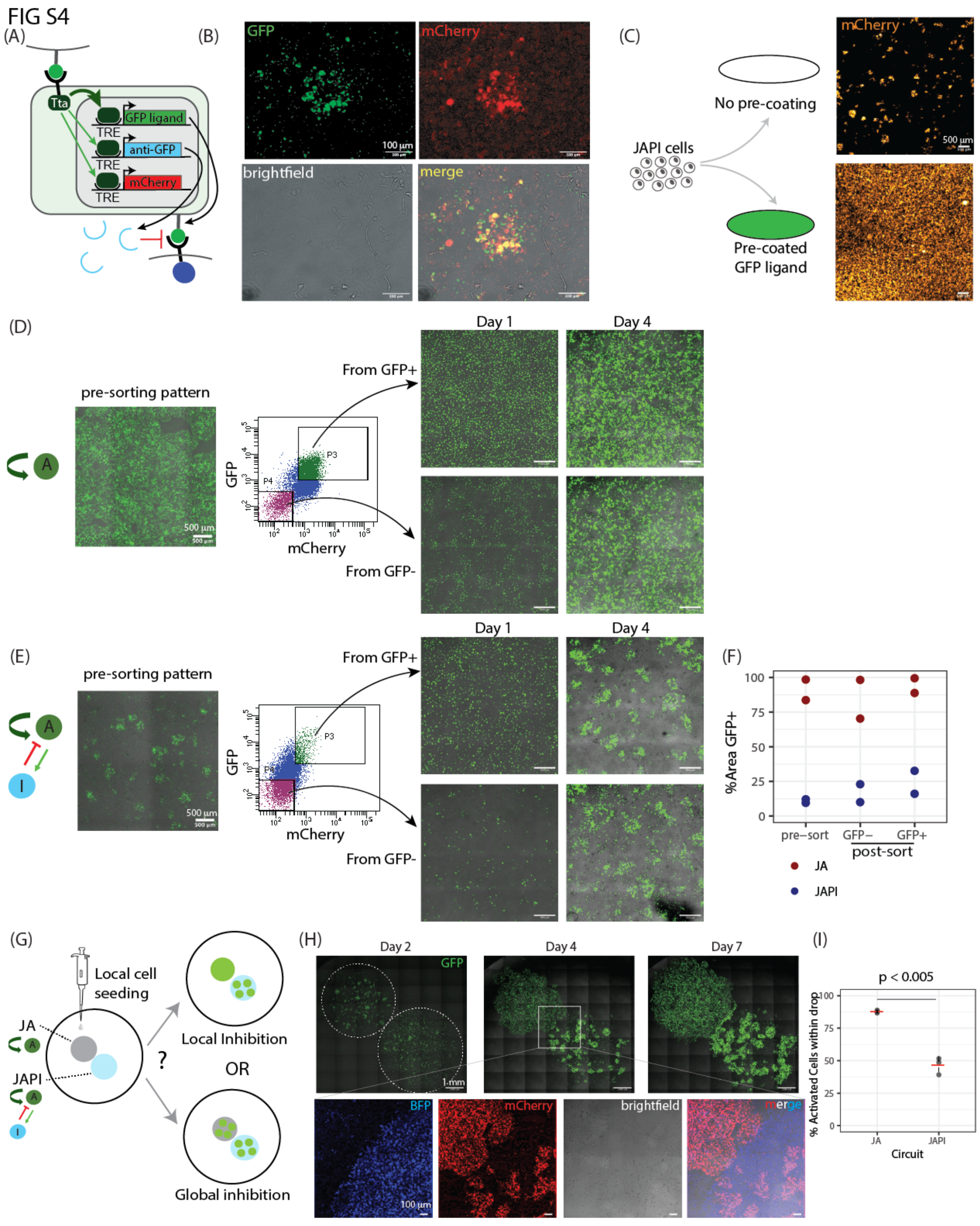

Fig. S4, related to Fig. 2. JAPI patterning reflects synNotch activity, is dynamically maintained, and arises from locally acting inhibition.

bioRxiv preprint doi: https://doi.org/10.64898/2026.05.24.727552; this version posted May 25, 2026. The copyright holder for this preprint (which was not certified by peer review) is the author/funder, who has granted bioRxiv a license to display the preprint in perpetuity. It is made available under a CC-BY 4.0 International license.

(**A**) Schematic of the JAPI circuit, modified with an additional intracellular mCherry reporter of synNotch activation. The anti-GFP-Tta synNotch receptor drives transcription from three TRE-promoted cassettes: the membrane-tethered GFP ligand (juxtacrine activator, green), the soluble anti-GFP nanobody dimer (paracrine inhibitor, light blue), and the cytoplasmic mCherry reporter (red). (**B**) Fluorescence microscope images of a clonal cell line containing the circuit from (A), showing the same field of view in four channels after 4 days of culture: GFP signal (top left, green), mCherry signal (top right, red), brightfield (bottom left, grey), and merge (bottom right). Initial condition: homogeneous inactivated cell lawn. Scale bar = 100 μm. (**C**) Test of whether JAPI cells retain the ability to activate from external ligand presentation. Left, schematic of the experimental setup: initially inactivated JAPI cells containing the circuit from (A) are plated at day zero either on a non-coated culture well (top) or on a culture well pre-coated with GFP ligand (bottom). Right, fluorescence microscope images at day four of each experiment, showing expression of the intracellular mCherry reporter (orange). Scale bar = 500 μm. (**D-E**) Pipeline of testing the reversibility of activation and inhibition for cells containing a JA (D) or JAPI (E) circuit, shown left to right as three stages: (i) the pre-sorting pattern at day 4 after onset of patterning, (ii) FACS sorting of the patterned cell population into activated (P3, GFP and mCherry double-positive) and inactivated (P4, GFP and mCherry double-negative) sub-populations, and (iii) re-plating and re-imaging of each sub-population at day 1 and day 4 after sorting. The circuit schematic (JA in D, JAPI in E) is shown at the far left. Re-plated images are labeled "From GFP+" (top, from P3) and "From GFP-" (bottom, from P4). Green indicates activated cells (GFP signal); brightfield in grey. Scale bar = 500 μm. (**F**) Dot-plot graph of the area fraction covered by GFP signal across the conditions in (D-E), for cells containing the JA circuit (red) or the JAPI circuit (blue). Each dot is an independent replicate (n = 2). Image processing in Methods, Image Analysis. (**G**) Schematic of the experimental design testing whether JAPI-mediated inhibition acts locally or at the well scale. Left, JA cells and JAPI cells are seeded as adjacent drops (gray and light blue circles respectively) in close proximity within a single well, with the rest of the well empty. Right, two possible outcomes drawn as cartoons of the well at a later timepoint: top, local inhibition, where JA cells in the JA drop activate completely and independently of the JAPI drop; bottom, global inhibition, where JAPI cells suppress JA cell activation in their drop. (**H**) Top, fluorescence microscope images at the indicated timepoints of a timelapse experiment of the setup in (G); the same field of view is shown across timepoints. Green indicates activated cells (GFP signal). Dotted lines on the day 2 image indicate the position of the two locally seeded cell drops (JA on top left, JAPI on bottom right). White rectangle on the day 4 image indicates the position of the zoom shown in the bottom panels. Bottom, zoom of the contact zone between the two cell drops at day 4, showing four channels of the same field of view: BFP (blue, marking the JAPI cells), mCherry (red, intracellular synNotch reporter in both JAPI and JA cells), brightfield (grey), and merge. (**I**) Dot-plot graph of the percentage of activated cells within each cell drop in the experiment of (H), separated by circuit (JA, JAPI) ,measured at day 4. Each dot is an individual replicate (n = 3); red bars indicate replicate means. p-values calculated from Welch's Two Sample t-test.

bioRxiv preprint doi: https://doi.org/10.64898/2026.05.24.727552; this version posted May 25, 2026. The copyright holder for this preprint (which was not certified by peer review) is the author/funder, who has granted bioRxiv a license to display the preprint in perpetuity. It is made available under a CC-BY 4.0 International license.

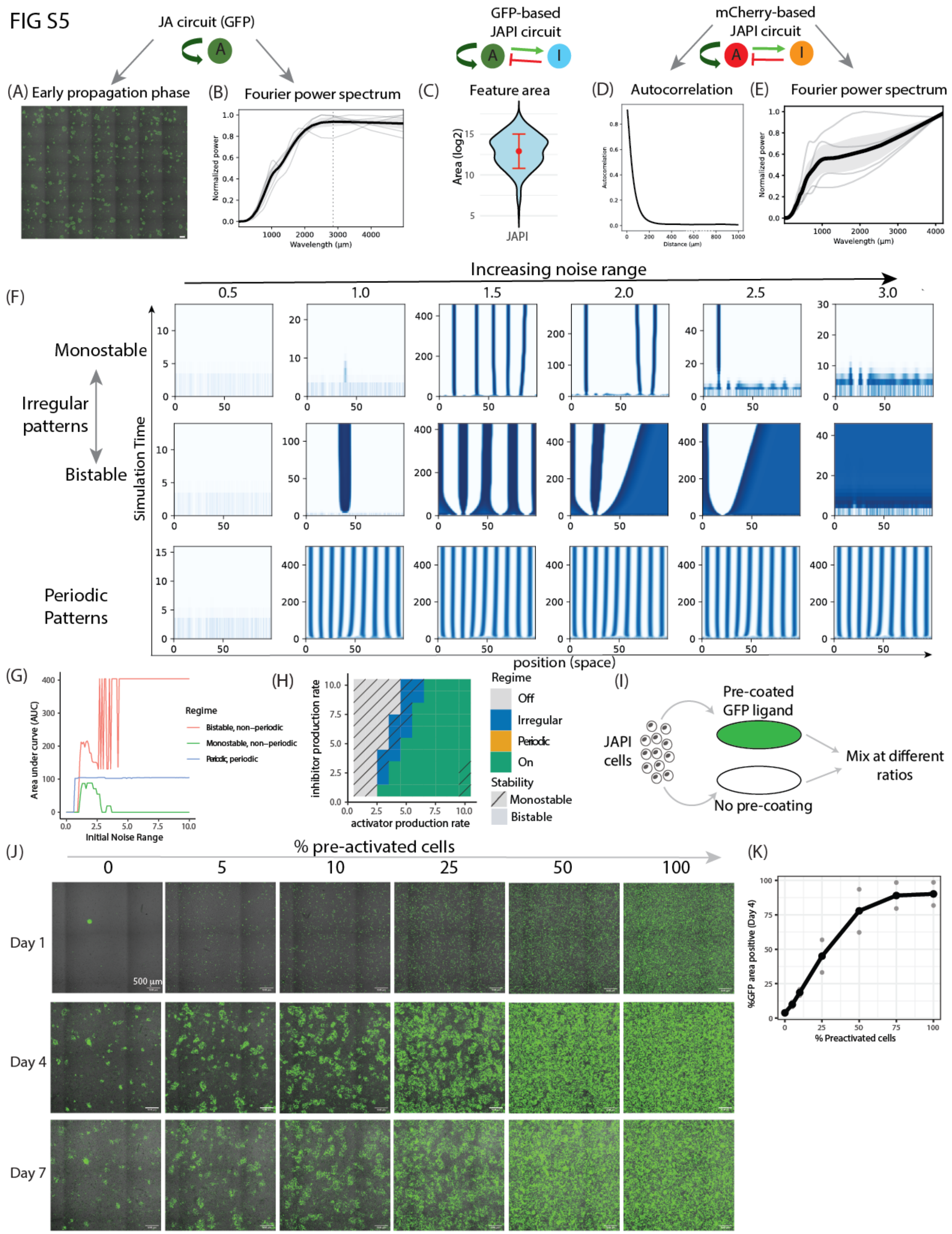

Fig. S5, related to Fig. 2. JAPI patterns show non-periodic spatial features and initial-condition sensitivity characteristic of the irregular regime.

bioRxiv preprint doi: https://doi.org/10.64898/2026.05.24.727552; this version posted May 25, 2026. The copyright holder for this preprint (which was not certified by peer review) is the author/funder, who has granted bioRxiv a license to display the preprint in perpetuity. It is made available under a CC-BY 4.0 International license.

(**A-B**) Representative microscope image and Fourier power spectrum of a cell line with the JA circuit depicted above. (A) Fluorescence microscope image of cells containing the GFP-based JA circuit at day 3, capturing the active propagation phase. Green indicates activated cells (GFP signal); brightfield in grey. Scale bar is 500 um. (B) Line plot of the Fourier power spectrum of processed GFP signal during the propagation phase, computed from images such as (A). Light grey curves are individual experiments, black curve is the average, grey shading is standard deviation (n = 5). Dotted vertical line marks an identified local maximum. Image processing in Methods, Image Analysis. (**C**) Violin plot of the distribution of activated domain areas measured from a clonal cell line containing the GFP-based JAPI circuit in the schematic above. Red dot indicates the mean, red bar indicates the standard deviation. Image processing in Methods, Image Analysis. (**D-E**) Statistical features of patterns measured at endpoint for a clonal cell line containing the mCherry-based circuit shown above and in Main Fig. 2E. (D) Line plot of the radially averaged autocorrelation of processed mCherry signal. Light grey curves are individual experiments, black curve is the average, grey shading is standard deviation (n = 4). (E) Line plot of the Fourier power spectrum of processed mCherry signal. Light grey curves are individual experiments, black curve is the average, grey shading is standard deviation (n = 4). Image processing in Methods, Image Analysis. (**F**) Grid of kymographs of one-dimensional simulations across three parameter regimes (rows, labeled at left: monostable irregular, bistable irregular, and periodic patterns) and six values of initial noise range (columns). The noise range n indicates the range of the random noise initial condition, as every cell receives an initial random activation value between 0 and n. Within each kymograph, x axis is one-dimensional position, y axis is simulation time progressing upward (note the time axis scale differs between panels to capture the relevant dynamics), and blue intensity indicates dimensionless activator concentration (see Supp. Note 3 for description of dimensionless units and stability regimes). Simulation setup and parameter values per each simulation is in Methods, Numerical Simulations. (**G**) Line plot of the area under the curve of the final activator concentration profile across position as a function of initial noise range, for the three parameter regimes shown in (F). Curves are color-coded by regime as indicated in the figure legend. Simulation setup in Methods, Numerical Simulations. (**H**) Phase diagram of regime classification for a JAPI circuit on the activator/inhibitor production rate axes, with an overlaid hatching pattern indicating monostable (single hatching) vs bistable (no hatching) regions. Tiles are colored by regime as indicated in the legend on the figure. Note that, for this parameter set, no combination of production rates generates periodic patterns, and that the irregular patterning regime spans both monostable and bistable parameter sets. Classification rule and bistability determination in Methods, Defining Patterning Regimes. (**I**) Schematic of the experimental design testing the sensitivity of JAPI patterning to initial conditions. Cells from a pre-coated GFP-ligand well (activated JAPI cells, green) and cells from a non-coated well (inactivated JAPI cells, white) are mixed at varying ratios and re-plated. (**J**) Fluorescence microscope images at the indicated timepoints of a timelapse experiment of the setup in (I); each column shows a different starting fraction of pre-activated cells; each row shows the same field of view across time. Green indicates activated cells (GFP signal); brightfield in grey. Scale bar = 500 µm. (**K**) Dot plot of the percentage of GFP-positive area at day 4 as a function of the percentage of pre-activated cells at time zero, from experiments as in (J). Each dot is an individual replicate (n = 2); black curve connects the means. Image processing in Methods, Image Analysis.

bioRxiv preprint doi: https://doi.org/10.64898/2026.05.24.727552; this version posted May 25, 2026. The copyright holder for this preprint (which was not certified by peer review) is the author/funder, who has granted bioRxiv a license to display the preprint in perpetuity. It is made available under a CC-BY 4.0 International license.

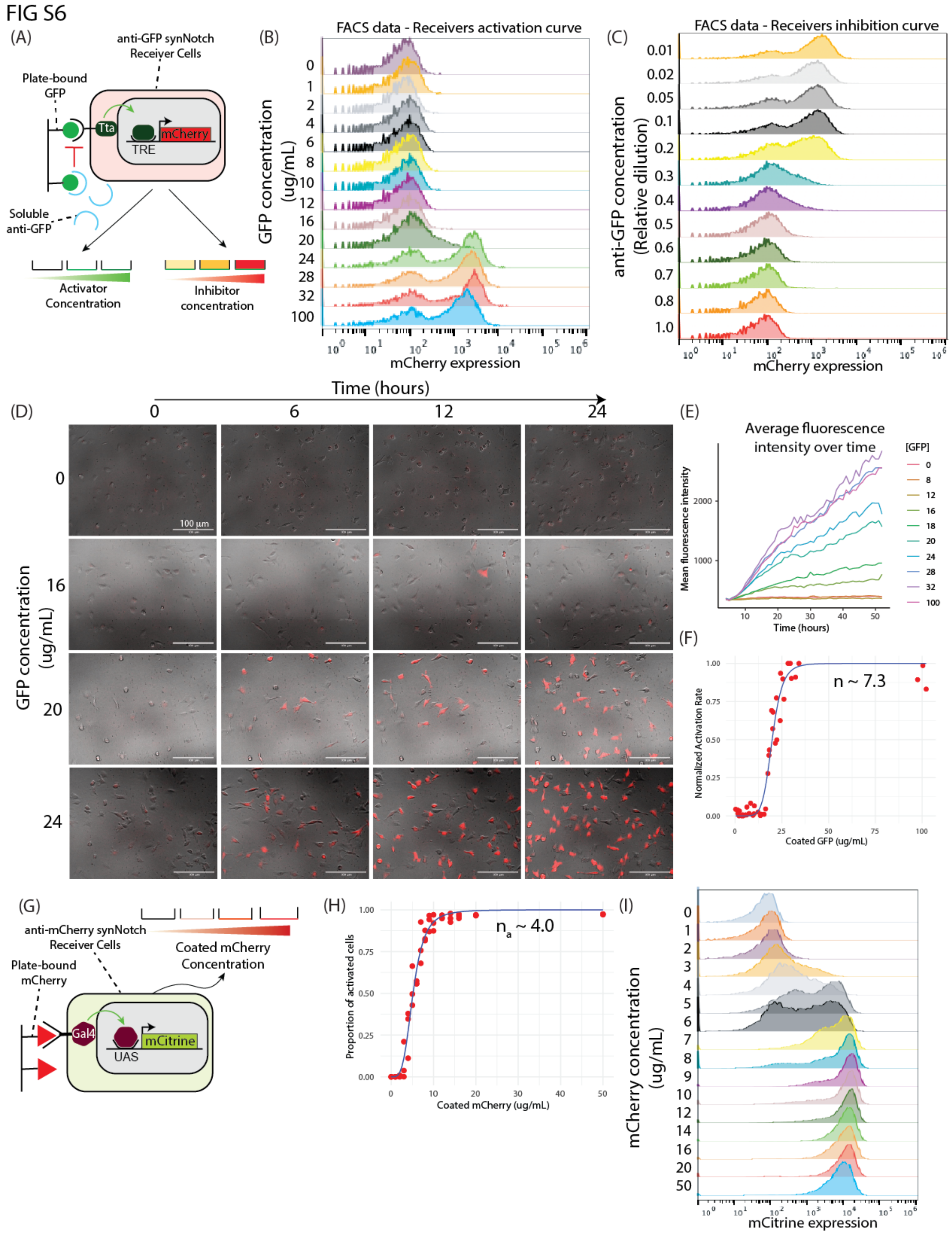

Fig. S6, related to Fig. 2. synNotch receptors show non-linear activation and inhibition, with Hill coefficients depending on the receptor architecture.

bioRxiv preprint doi: https://doi.org/10.64898/2026.05.24.727552; this version posted May 25, 2026. The copyright holder for this preprint (which was not certified by peer review) is the author/funder, who has granted bioRxiv a license to display the preprint in perpetuity. It is made available under a CC-BY 4.0 International license.

(**A**) Schematic of the experimental setup for measuring synNotch dose-response curves. A receiver cell line constitutively expressing an anti-GFP-Tta synNotch receptor with a downstream TRE-promoted mCherry reporter is plated at low confluency under two conditions: left, on a culture well coated with a varying concentration of GFP ligand (activator) to measure activation; right, in culture medium containing a varying concentration of soluble anti-GFP inhibitor in the presence of constant activator concentration, to measure inhibition. In both cases, the output of activation is read as mCherry expression. (**B**) FACS histograms of mCherry expression in receiver cells from (A) 48 hours after plating on increasing concentrations of plate-bound GFP ligand (rows, labeled at left). (**C**) FACS histograms of mCherry expression in receiver cells from (A) 48 hours after plating on a constant concentration of plate-bound GFP ligand and increasing concentrations of soluble anti-GFP inhibitor (rows, labeled at left, in relative dilution of inhibitor-containing conditioned media). (**D**) Fluorescence microscope images at the indicated timepoints of a timelapse experiment of receiver cells from (A) plated on increasing concentrations of plate-bound GFP ligand (rows, labeled at left). Red indicates synNotch-activated cells (mCherry reporter); brightfield in grey. Scale bar = 500 µm. (**E**) Line plot of mean mCherry fluorescence intensity over time from the experiments in (D), for each plate-bound GFP concentration (curves color-coded by concentration as indicated in the figure legend). Curves indicate the means of n = 3 independent experiments. Image processing in Methods, Image Analysis. (**F**) Scatter plot with fitted Hill curve of normalized activation rate as a function of plate-bound GFP concentration, computed from the slopes of the curves in (E). Red dots are individual experimental data points from n = 3 independent replicates; blue curve is the fitted activating Hill function. The fitted Hill coefficient is indicated in the figure. Fitting procedure in Methods, synNotch parametrization experiments. (**G**) Schematic of the experimental setup for measuring dose-response curves of a second synNotch receptor with an orthogonal architecture constructed with a mCherry ligand binding domain and Gal4 intracellular transactivation domain. A receiver cell line constitutively expressing an anti-mCherry-Gal4 synNotch receptor with a downstream UAS-promoted mCitrine reporter is plated on a culture well coated with mCherry ligand (varying concentration). The output of activation is read as mCitrine expression. (**H**) Scatter plot with fitted Hill curve of the proportion of activated receiver cells from (G) as a function of plate-bound mCherry concentration, computed from FACS data such as shown in (I). Red dots are individual experimental data points from n = 3 independent experiments; blue curve is the fitted activating Hill function. The fitted Hill coefficient is indicated in the figure. Fitting in Methods, synNotch parametrization experiments. (**I**) FACS histograms of mCitrine expression in receiver cells from (G) plated on increasing concentrations of plate-bound mCherry ligand (rows, labeled at left, in µg/mL).

bioRxiv preprint doi: https://doi.org/10.64898/2026.05.24.727552; this version posted May 25, 2026. The copyright holder for this preprint (which was not certified by peer review) is the author/funder, who has granted bioRxiv a license to display the preprint in perpetuity. It is made available under a CC-BY 4.0 International license.

FIG S7

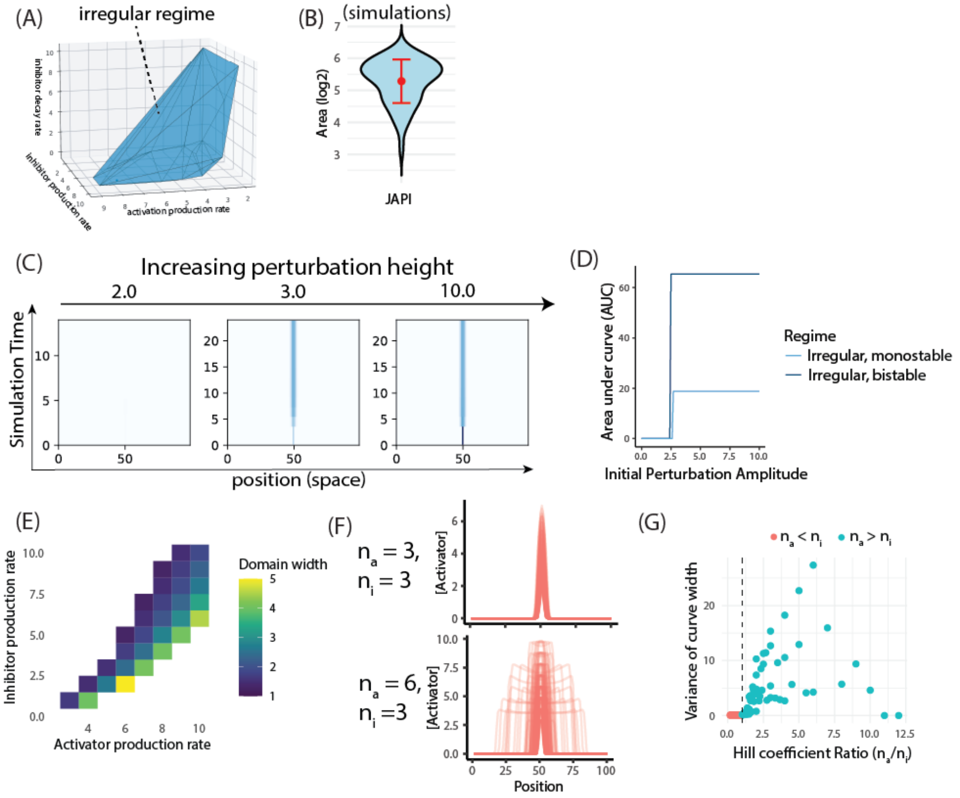

Fig. S7, related to Fig. 2. Domain size in the irregular regime is set by activator-inhibitor balance and depends on relative activator and inhibitor Hill cooperativity.

(**A**) 3D plot showing the volume of parameter space within the irregular regime for a JAPI circuit with parametrized Hill coefficients (n_a = 10, n_i = 4). Axes are the three remaining non-dimensionalized parameters: activator production rate β_a, inhibitor production rate β_i, and inhibitor degradation rate γ. Blue points mark parameter combinations classified as irregular; non-blue points mark homogeneous regimes (all on or all off). Classification rule in Methods, Defining Patterning Regimes. (**B**) Violin plot of the distribution of feature area from parametrized JAPI simulations (n_a = 10, n_i = 4), initiated from continuous stochastic activation bursts. Red dot indicates the mean, red bar indicates the standard deviation (n = 3 replicated simulations). Compare to S5A, which shows the same metric measured experimentally. Simulation setup in Methods, Numerical Simulations. (**C**) Kymographs of one-dimensional JAPI simulations initiated from a centrally located activator burst of increasing amplitude (columns), for a parameter set within the irregular regime. Blue intensity indicates dimensionless activator concentration (see Supp. Note 3 for description of dimensionless units). Simulation setup and parameter values per each simulation is in Methods, Numerical Simulations. (**D**) Line plot of the area under the curve of the final activator concentration profile across position as a function of initial perturbation amplitude, for two parameter sets within the irregular regime: monostable (light blue) and bistable (dark blue). Simulation setup in Methods, Numerical Simulations. (**E**) Heatmap of the width of the stable activated domain emerging from a centrally located activation burst, as a function of activator production rate (x axis) and inhibitor production rate (y axis), with the remaining non-dimensionalized parameters held constant ($n_a = 6$, $n_i = 3$, $\gamma = 0.1$) within the irregular regime. Color represents domain width quantified by the standard deviation of the activator spatial distribution, expressed in cell units (see colorbar). Simulation setup and parameter values in Methods,

bioRxiv preprint doi: https://doi.org/10.64898/2026.05.24.727552; this version posted May 25, 2026. The copyright holder for this preprint (which was not certified by peer review) is the author/funder, who has granted bioRxiv a license to display the preprint in perpetuity. It is made available under a CC-BY 4.0 International license.

Numerical Simulations. (**F**) Line plot of the activator concentration profile across one-dimensional position, overlaying the outcome of ~160 simulations from a centrally located activation burst across varying activator and inhibitor production rates and inhibitor degradation rate, for two pairs of Hill coefficients shown separately. Each light red curve is an individual parameter combination outcome; the spread illustrates the parameter sensitivity of domain width. Simulation setup and parameter values in Methods, Numerical Simulations. (**G**) Scatter plot of the variance of activator peak width across parameter sets within the irregular regime as a function of the Hill coefficient ratio n_a / n_i (n = 12 x 12 = 144 total points). Red dots, parameter sets with $n_a < n_i$; teal dots, parameter sets with $n_a > n_i$. Dashed vertical line marks the $n_a / n_i = 1$ boundary. Simulation setup and parameter values in Methods, Numerical Simulations.

bioRxiv preprint doi: https://doi.org/10.64898/2026.05.24.727552; this version posted May 25, 2026. The copyright holder for this preprint (which was not certified by peer review) is the author/funder, who has granted bioRxiv a license to display the preprint in perpetuity. It is made available under a CC-BY 4.0 International license.

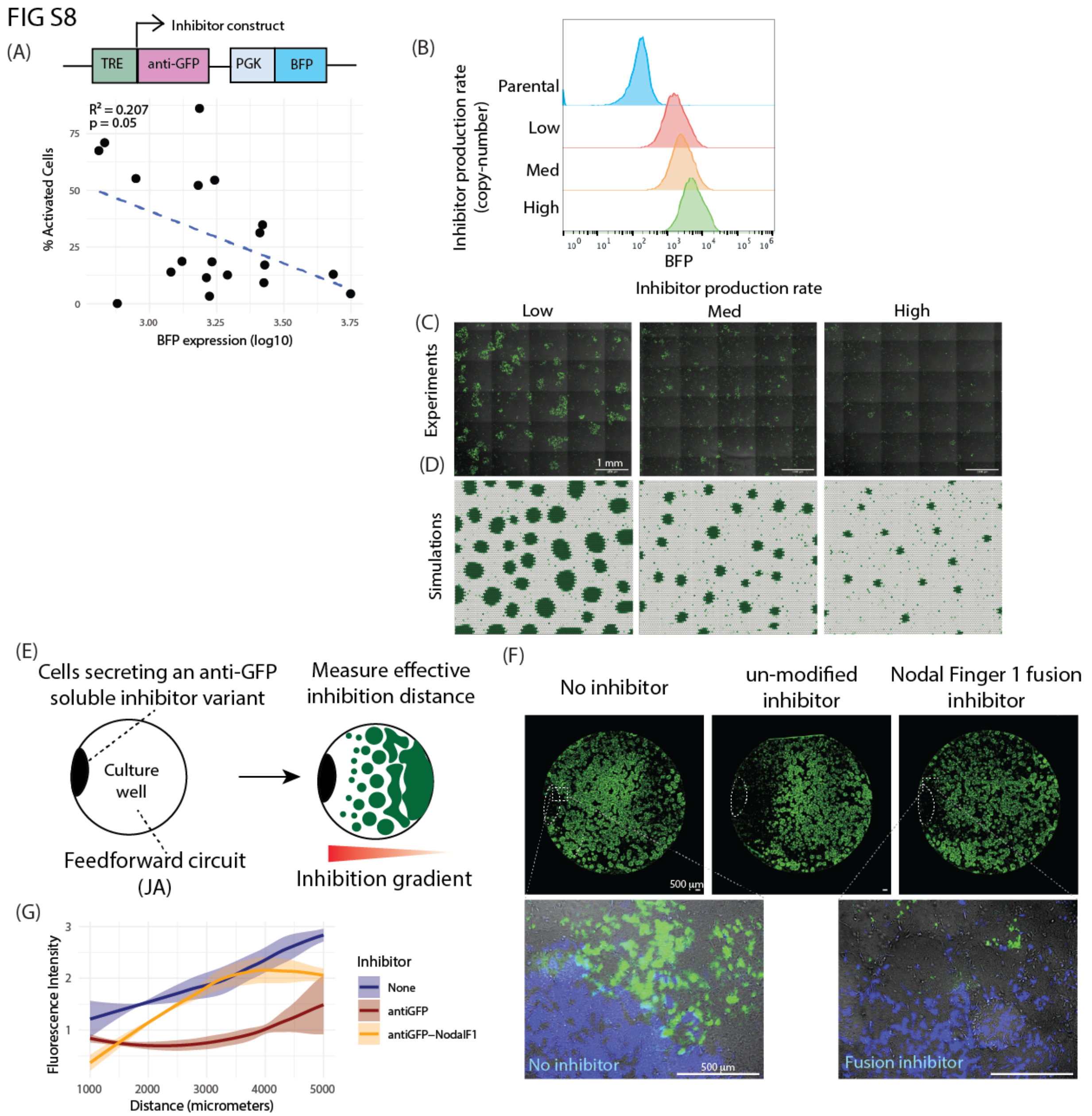

Fig. S8, related to Fig. 3. Validation of inhibitor production rate and diffusion length as valid parameter knobs for tuning domain size in JAPI-mediated irregular patterning.
(**A**) Top, schematic of the lentiviral construct used to add downstream activation of a paracrine inhibitor in a JAPI circuit. Bottom, scatter plot with linear regression of the percentage of activated cells as a function of BFP expression (log10 scale). Each dot is a measurement made from an individual clonal line (n = 19); dashed line is the best-fit linear regression. R^2 and p-value indicated in the figure. (**B**) FACS histograms of BFP expression in three polyclonal cell lines bulk-sorted from a population of JAPI cells based on distinct BFP expression profiles (rows, labeled at left). (**C**) Fluorescence microscope endpoint images at day 4 of the three bulk-sorted JAPI cell lines from (B), with increasing inhibitor production rate β_i (left to right). Green indicates activated cells (GFP signal); brightfield in grey. Initial condition: homogeneous inactivated cell lawn. Scale bar = 500 µm. Quantification of these data is shown in Fig. 3C. (**D**) Endpoint snapshots of two-

bioRxiv preprint doi: https://doi.org/10.64898/2026.05.24.727552; this version posted May 25, 2026. The copyright holder for this preprint (which was not certified by peer review) is the author/funder, who has granted bioRxiv a license to display the preprint in perpetuity. It is made available under a CC-BY 4.0 International license.

dimensional JAPI simulations parametrized with the Hill coefficients from Fig. 2J-K (n_a = 10, n_i = 4), with three increasing values of the inhibitor production rate β_i (left to right). Green intensity indicates dimensionless activator concentration (see Supp. Note 3 for description of dimensionless units). Simulation setup and parameter values per each simulation is in Methods, Numerical Simulations. Quantification of these data is shown in Main Fig. 3D. (**E**) Schematic of the experimental setup for measuring the effective diffusion length of paracrine anti-GFP inhibitor variants. Left, cells constitutively secreting a soluble anti-GFP inhibitor variant are locally seeded as a drop at the left edge of the well, and the rest of the well is plated with JA cells. Right, cartoon of the expected outcome inside the well at a later timepoint: the inhibitor-secreting drop on the left edge, a graded distribution of activated GFP cells (green) across the well with fewer activated cells closer to the source. The inferred underlying inhibitor gradient is abstractly depicted as the red triangle at the bottom. (**F**) Fluorescence microscope endpoint images at day 4 of the setup in (E), for three conditions in columns (labeled at top). No inhibitor corresponds to a condition where parental cells were locally seeded. Top row, full-well view; bottom row, zoom of the indicated regions showing the boundary between the locally seeded cells (BFP, blue, expressed in all three locally seeded lines as a tracking marker) and activation of the JA cells (GFP, green). Scale bar = 500 μm. (**G**) Line plot of normalized fluorescence intensity of the JA-cell GFP signal as a function of distance from the locally seeded drop, for the three conditions in (F). Curves are means of independent replicates with shading for standard deviation (n = 3). Image processing in Methods, Image Analysis.

bioRxiv preprint doi: https://doi.org/10.64898/2026.05.24.727552; this version posted May 25, 2026. The copyright holder for this preprint (which was not certified by peer review) is the author/funder, who has granted bioRxiv a license to display the preprint in perpetuity. It is made available under a CC-BY 4.0 International license.

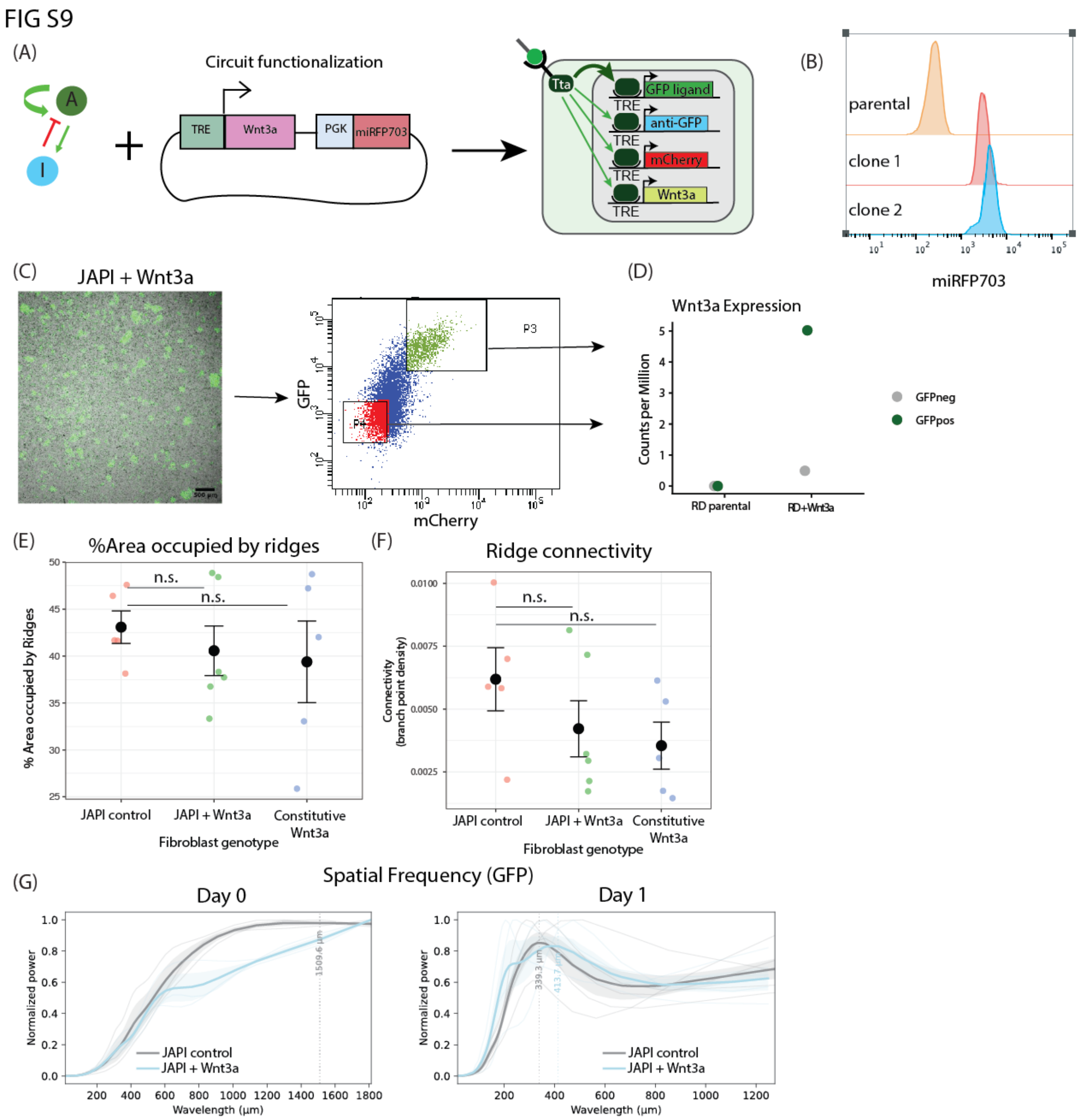

Fig. S9, related to Fig. 3. Functionalization of a JAPI circuit for downstream patterned production of Wnt3a, and characterization of their interaction with dorsal chicken embryonic epidermis.
(**A**) Schematic of the functionalization of a JAPI circuit by addition of a Wnt3a expression cassette downstream of synNotch activation, showing the lentiviral construct added to the circuit. Right, the resulting functionalized JAPI circuit with four cassettes downstream of synNotch receptor activation. (**B**) FACS histograms of miRFP703 expression in three cell populations (rows, labeled at left): parental JAPI cells, and two clonal lines generated from infection with the construct in (A). (**C**) Left, fluorescence microscope image and FACS gating strategy for transcriptomic analysis of clonal cells containing a functionalized JAPI circuit. Green indicates activated cells (GFP signal); brightfield in grey. Scale bar = 500 µm. Right, FACS plot showing the gating used to sort activated (P3, GFP and mCherry double-positive) and inactivated (P5, GFP and mCherry

bioRxiv preprint doi: https://doi.org/10.64898/2026.05.24.727552; this version posted May 25, 2026. The copyright holder for this preprint (which was not certified by peer review) is the author/funder, who has granted bioRxiv a license to display the preprint in perpetuity. It is made available under a CC-BY 4.0 International license.

double-negative) cell populations for transcriptomic profiling by RNA-seq. (**D**) Dot plot of Wnt3a transcript expression measured by RNA-seq, for the two sorted populations from (C) ("RD + Wnt3a") in comparison to sorted populations from parental JAPI cells (no Wnt3a transgene, "RD parental"). Each dot is an individual replicate (n = 1). (**E**) Dot plot of the percentage of epithelial area covered by ridges 24 hours after recombination, separated by underlying engineered fibroblast genotype. Each dot is an individual replicate; black bars indicate mean and standard deviation (n = 5). p-values (n.s. = not significant) calculated from Welch's Two Sample t-test. Image processing in Methods, Chicken recombination experiments. (**F**) Scatter plot of the ridge connectivity (branch-point density on a skeletonized binary image) of the epithelial ridges 24 hours after recombination, separated by underlying engineered fibroblast genotype. Each dot is an individual replicate (n = 5); black bars indicate mean and standard deviation. p-values (n.s. = not significant) calculated from Welch's Two Sample t-test. Skeletonization procedure in Methods, Chicken recombination experiments. (**G**) Line plot of the Fourier power spectrum of the GFP signal of JAPI control and JAPI + Wnt3a engineered fibroblasts, shown at day 0 (left, before recombination) and day 1 (right, 24 hours after recombination with embryonic dorsal chicken epithelium). Light grey curves are individual experiments, dark curves are the average for each condition, shading is standard deviation (n = 5 for JAPI control, n = 3 for PAI + Wnt3a). Dotted vertical lines mark identified local maxima. Image processing in Methods, Chicken recombination experiments.

bioRxiv preprint doi: https://doi.org/10.64898/2026.05.24.727552; this version posted May 25, 2026. The copyright holder for this preprint (which was not certified by peer review) is the author/funder, who has granted bioRxiv a license to display the preprint in perpetuity. It is made available under a CC-BY 4.0 International license.

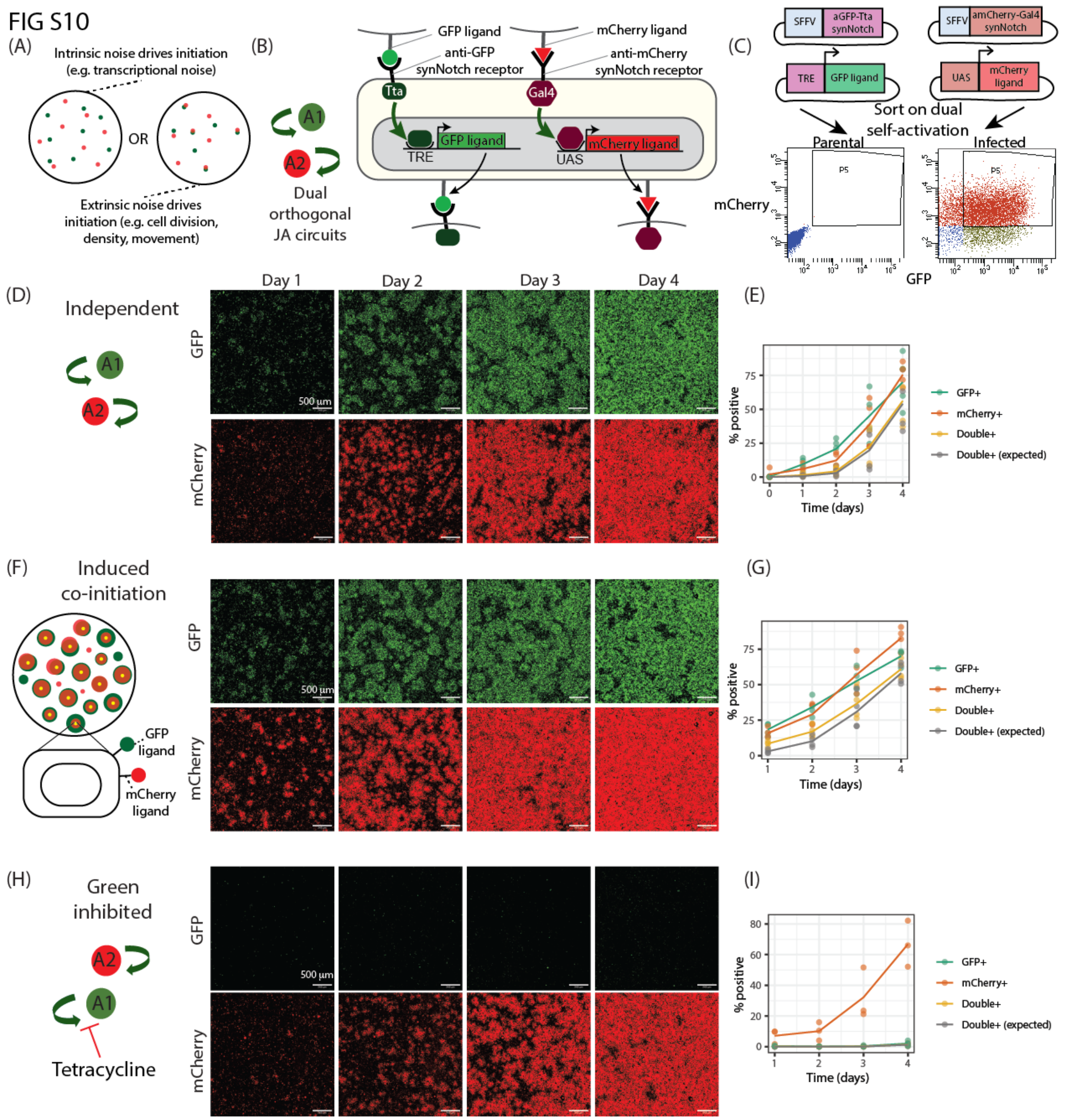

Fig. S10, related to Fig. 4. Construction and characterization of dual orthogonal JA circuits in a single cell line.

(**A**) Schematic of two possible outcomes for the initiation of dual orthogonal synNotch-based juxtacrine activation (JA) circuits, drawn as cartoons of a cell well at an early timepoint. Left, intrinsic noise drives initiation: each circuit initiates independently from transcriptional noise (green and red dots in distinct cells). Right, extrinsic noise drives initiation: shared cellular events (cell division, density, movement) co-initiate signal propagation of both circuits in the same cells. (**B**) Schematic of a circuit encoding dual orthogonal synNotch-mediated juxtacrine self-activation (JA), without paracrine inhibition. The anti-GFP-Tta synNotch receptor drives TRE-promoted GFP ligand; the anti-mCherry-Gal4 synNotch receptor drives UAS-promoted mCherry ligand. The two circuits are theoretically completely orthogonal. (**C**) Infection strategy for generating the dual-

bioRxiv preprint doi: https://doi.org/10.64898/2026.05.24.727552; this version posted May 25, 2026. The copyright holder for this preprint (which was not certified by peer review) is the author/funder, who has granted bioRxiv a license to display the preprint in perpetuity. It is made available under a CC-BY 4.0 International license.

JA cell line. Top, schematic of the four lentiviral constructs used: two SFFV-promoted synNotch receptors (anti-GFP-Tta, anti-mCherry-Gal4) and two response-promoted ligands (TRE-GFP ligand, UAS-mCherry ligand). Bottom, FACS plots showing the sorting strategy: parental cells (left, baseline) and infected cells (right, post-infection); the P5 gate marks cells positive for both GFP and mCherry, used for single-cell sorting to generate clonal dual-JA lines. (**D**) Fluorescence microscope images at the indicated timepoints of a timelapse experiment of cells containing the dual-JA circuit from (B); the same field of view is shown across timepoints. Scale bar = 500 µm. The merge of the two channels is shown in Fig. 4C. (**E**) Plot of the percentage of single and dual activated cells over time, measured by FACS from the experiment in (D). Curves color-coded by population, with grey representing double positive cells expected by independent probability. Each dot is an individual replicate (n = 4); lines connect replicate means. (**F**) Left, schematic of the induced co-initiation experiment: dual-JA cells from (B) are mixed with 1% cells constitutively expressing both GFP and mCherry ligands (dual senders). Right, fluorescence microscope images at the indicated timepoints of a timelapse experiment of this setup; the same field of view is shown across timepoints. Scale bar = 500 µm. The merge of the two channels is shown in Fig. 4E. (**G**) Plot of the percentage of single and dual activated cells over time, measured by FACS from the experiment in (F) (n = 4). Curves color-coded by population as in (E). (**H**) Fluorescence microscope images at the indicated timepoints of a timelapse experiment of the dual-JA cells from (D) in medium containing tetracycline, which inhibits the Tta domain of the anti-GFP synNotch receptor and selectively blocks the GFP-based circuit; the same field of view is shown across timepoints. Scale bar = 500 µm. The merge of the two channels is shown in Fig. 4G. (**I**) Line plot of the percentage of GFP-positive, mCherry-positive, and double-positive cells over time, measured by FACS from the experiment in (H) (n = 3). Curves color-coded by population as in (E).

bioRxiv preprint doi: https://doi.org/10.64898/2026.05.24.727552; this version posted May 25, 2026. The copyright holder for this preprint (which was not certified by peer review) is the author/funder, who has granted bioRxiv a license to display the preprint in perpetuity. It is made available under a CC-BY 4.0 International license.

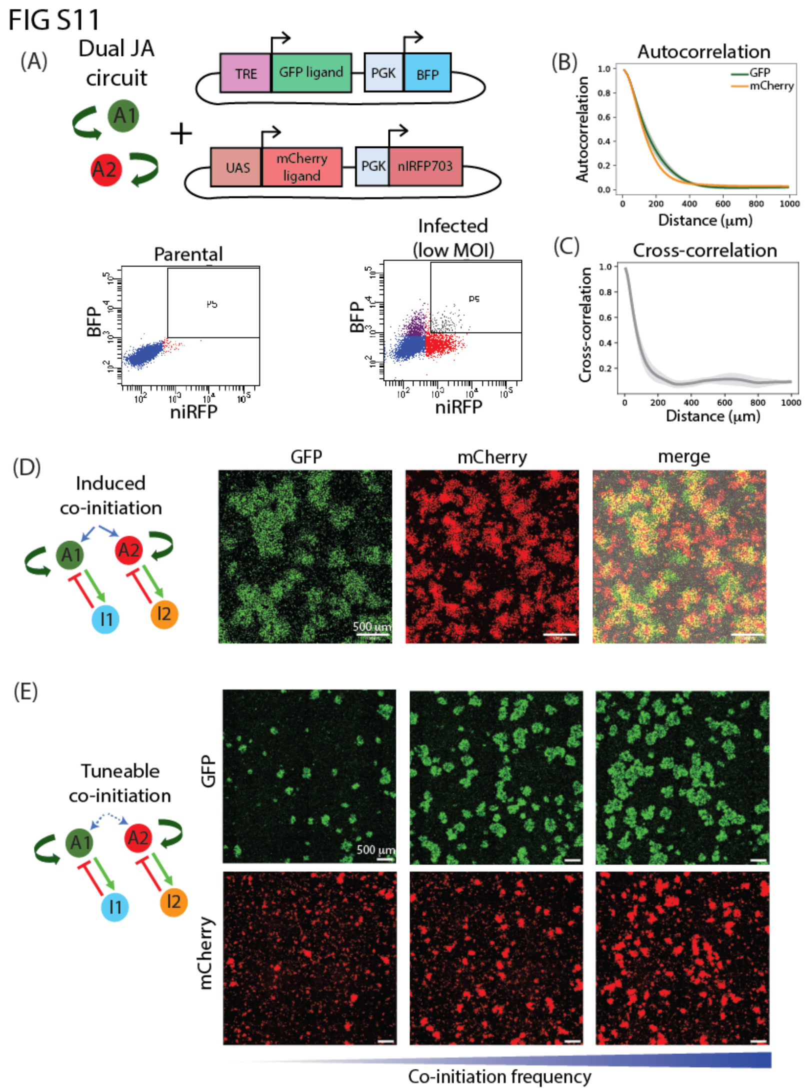

Fig. S11, related to Fig. 4. Construction and characterization of dual orthogonal JAPI circuits in a single cell line. (**A**) Top, schematic of the implementation of dual orthogonal JAPI circuits by adding two inducible inhibitor transgenes to the dual-JA cell line from (S11B). Left, the parental dual-JA circuit. Right, the two added lentiviral constructs. Bottom, FACS sorting strategy for dual-JA cells receiving both inhibitor modules. The P5 gate marks cells positive for both BFP and niRFP, sorted to generate clonal dual-JAPI lines. (**B**) Line plot of the radially averaged autocorrelation of GFP signal (green) and mCherry signal (orange) from patterns generated by a dual-JAPI cell line. Curves are means with shading for standard deviation across replicates (n = 3). Image processing in Methods, Image Analysis. (**C**) Line plot of the radially averaged cross-correlation between GFP and mCherry signals from patterns generated by a dual-JAPI cell line. Curve is the mean with shading for standard deviation across replicates (n = 3). Image processing in Methods, Image Analysis. (**D**) Single-channel fluorescence microscope endpoint images at day 4 of cells containing dual orthogonal JAPI circuits mixed with 1% of cells constitutively expressing ligand GFP and ligand mCherry (dual senders). (**E**) Single-channel fluorescence microscope endpoint images at day 4 of cells containing dual orthogonal JAPI circuits mixed with increasing numbers of dual senders (left to right). Each image shows the separate channels corresponding to the merged images shown in Fig 4N. Scale bar = 500 μm.

bioRxiv preprint doi: https://doi.org/10.64898/2026.05.24.727552; this version posted May 25, 2026. The copyright holder for this preprint (which was not certified by peer review) is the author/funder, who has granted bioRxiv a license to display the preprint in perpetuity. It is made available under a CC-BY 4.0 International license.

FIG S12

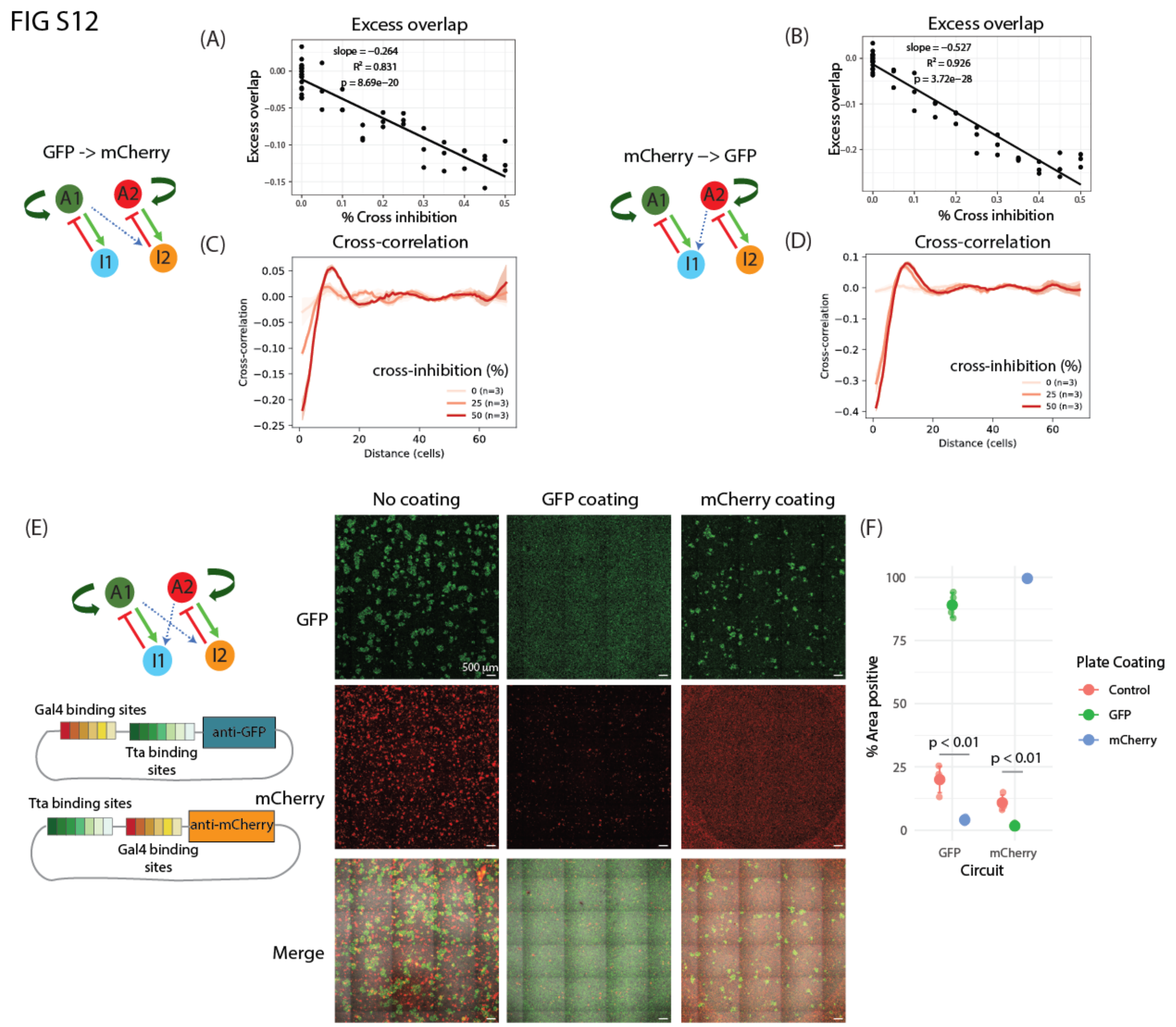

Fig. S12, related to Fig. 5. Characterization of uni-directional and bi-directional cross-inhibition in dual-JAPI circuits.
(**A-D**) Simulated uni-directional cross-inhibition. Left of the results panels, schematic of the dual JAPI circuit with uni-directional cross-inhibition GFP → mCherry, indicated by a dashed blue arrow. (**A**) Scatter plot with linear regression of excess overlap as a function of cross-inhibition strength, computed from two-dimensional simulation. Each dot is an individual simulation (n = 3 per condition); black line is the best-fit linear regression. Slope, R^2, and p-value indicated in the figure. Simulation setup and parameter values in Methods, Numerical Simulations. (**B**) Same as (A) for uni-directional cross-inhibition in the opposite direction (mCherry → GFP). (**C**) Line plot of the radially averaged cross-correlation between GFP and mCherry signals, for three representative cross-inhibition strengths from the simulations in (A) (curves color-coded by strength as indicated in the figure legend). Curves are means with shading for standard deviation across replicates (n = 3 per condition). Image processing in Methods, Image Analysis. (**D**) Same as (C) at three matching representative cross-inhibition strengths for uni-directional cross-inhibition in the opposite direction (mCherry → GFP). (**E**) Left, schematic of the bi-directional cross-inhibiting dual-JAPI circuit design. The promoter of each paracrine inhibitor cassette is built as a tandem of the two synNotch-inducible promoter elements (TRE and UAS), with the binding

bioRxiv preprint doi: https://doi.org/10.64898/2026.05.24.727552; this version posted May 25, 2026. The copyright holder for this preprint (which was not certified by peer review) is the author/funder, who has granted bioRxiv a license to display the preprint in perpetuity. It is made available under a CC-BY 4.0 International license.

sites of the cross-inhibiting synNotch placed further from the transcription start site than the cognate sites. Right, fluorescence microscope endpoint images for cells plated on culture wells with three coating conditions (no coating, GFP coating, mCherry coating), in order to force activation of one circuit and assess the consequence of cross-inhibition on the opposing signal. (**F**) Dot plot of the percentage of area positive for each fluorescent signal, separated by coating condition and fluorescent circuit reported. Dot colors encode plate coating condition. Each dot is an individual replicate (n = 3). p-values calculated using Welch's Two Sample t-test.

bioRxiv preprint doi: https://doi.org/10.64898/2026.05.24.727552; this version posted May 25, 2026. The copyright holder for this preprint (which was not certified by peer review) is the author/funder, who has granted bioRxiv a license to display the preprint in perpetuity. It is made available under a CC-BY 4.0 International license.

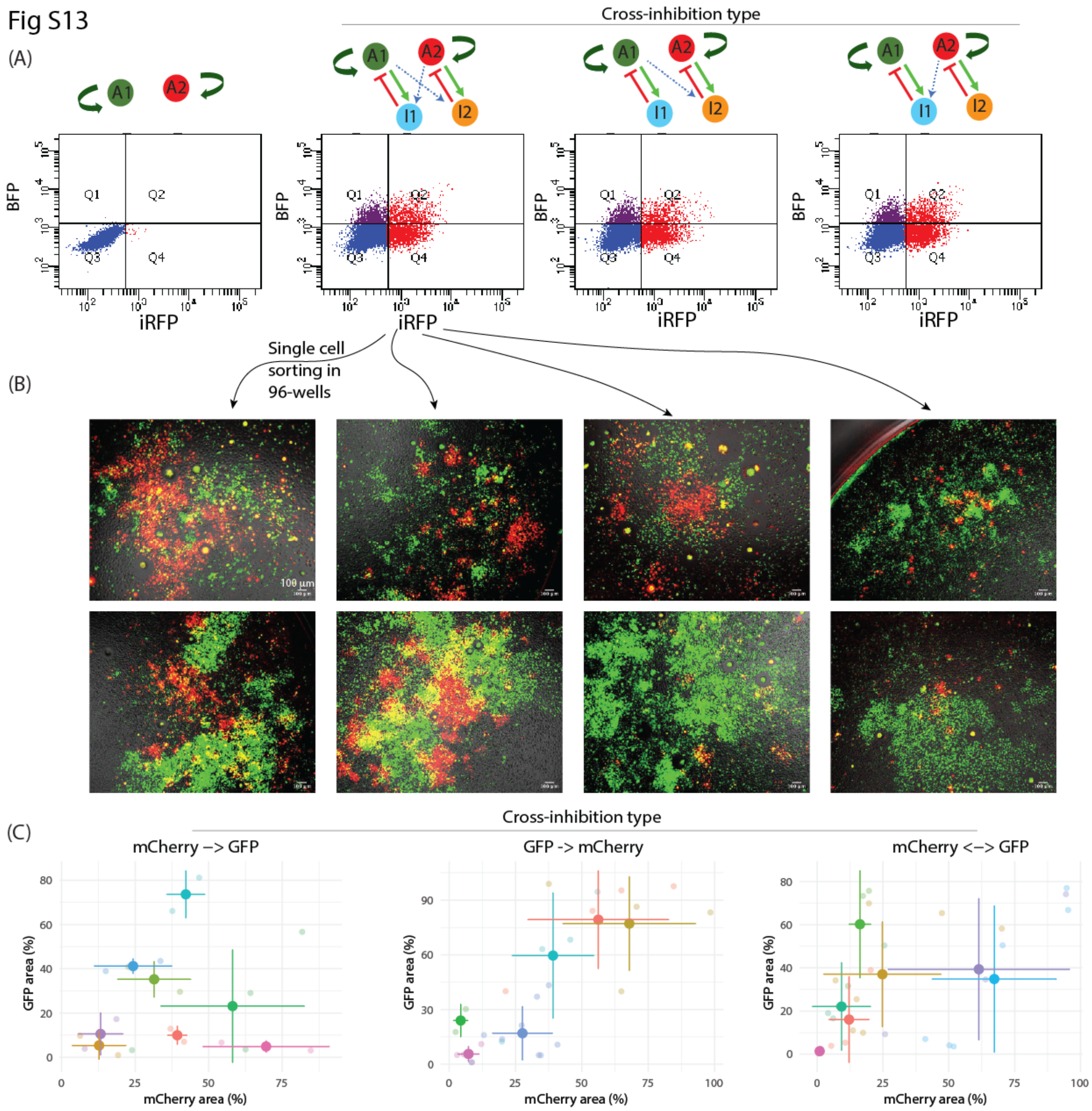

Fig. S13, related to Fig. 5. Clonal characterization of the cross-inhibiting dual-JAPI library. (**A**) FACS sorting strategy for generating clonal dual-JAPI cell lines with bi-directional or uni-directional cross-inhibition. Four plots are shown, where quadrant Q2 was used to generate clonal populations through single-cell sorting of dual reporter-positive cells in 96-well plates. Left, parental dual-JA cells (BFP-negative, iRFP-negative baseline). The next three plots show dual-JA cells infected at low MOI (MOI < 0.5) with three types of cross-inhibition library constructs (schematic above each plot): bi-directional cross-inhibition, uni-directional GFP → mCherry, and uni-directional mCherry → GFP. (**B**) Fluorescence microscope images of eight representative clonal cross-inhibiting dual-JAPI cell lines derived from the sorting in (A), taken in the same 96-well well around two weeks after single-cell sorting. Green indicates activated cells (GFP signal); red indicates activated cells (mCherry signal); brightfield in grey. Scale bar = 100 µm. (**C**) Scatter plot of the percentage of area covered by mCherry signal (x axis) versus the percentage of area covered by GFP signal (y axis), measured at day 4 across clonal dual-JAPI cell lines, separated by

bioRxiv preprint doi: https://doi.org/10.64898/2026.05.24.727552; this version posted May 25, 2026. The copyright holder for this preprint (which was not certified by peer review) is the author/funder, who has granted bioRxiv a license to display the preprint in perpetuity. It is made available under a CC-BY 4.0 International license.

cross-inhibition type. Three sub-panels are shown, from left to right: mCherry → GFP (n = 18 replicates across n = 8 clonal cell lines), GFP → mCherry (n = 27 replicates across n = 6 clonal cell lines), mCherry ↔ GFP (bi-directional; n = 29 replicates across n = 7 clonal cell lines). Dot colors group replicates by clonal cell line; bold dot with crosshairs indicates the mean and standard deviation across replicates for each clone. Image processing in Methods, Image Analysis.

bioRxiv preprint doi: https://doi.org/10.64898/2026.05.24.727552; this version posted May 25, 2026. The copyright holder for this preprint (which was not certified by peer review) is the author/funder, who has granted bioRxiv a license to display the preprint in perpetuity. It is made available under a CC-BY 4.0 International license.

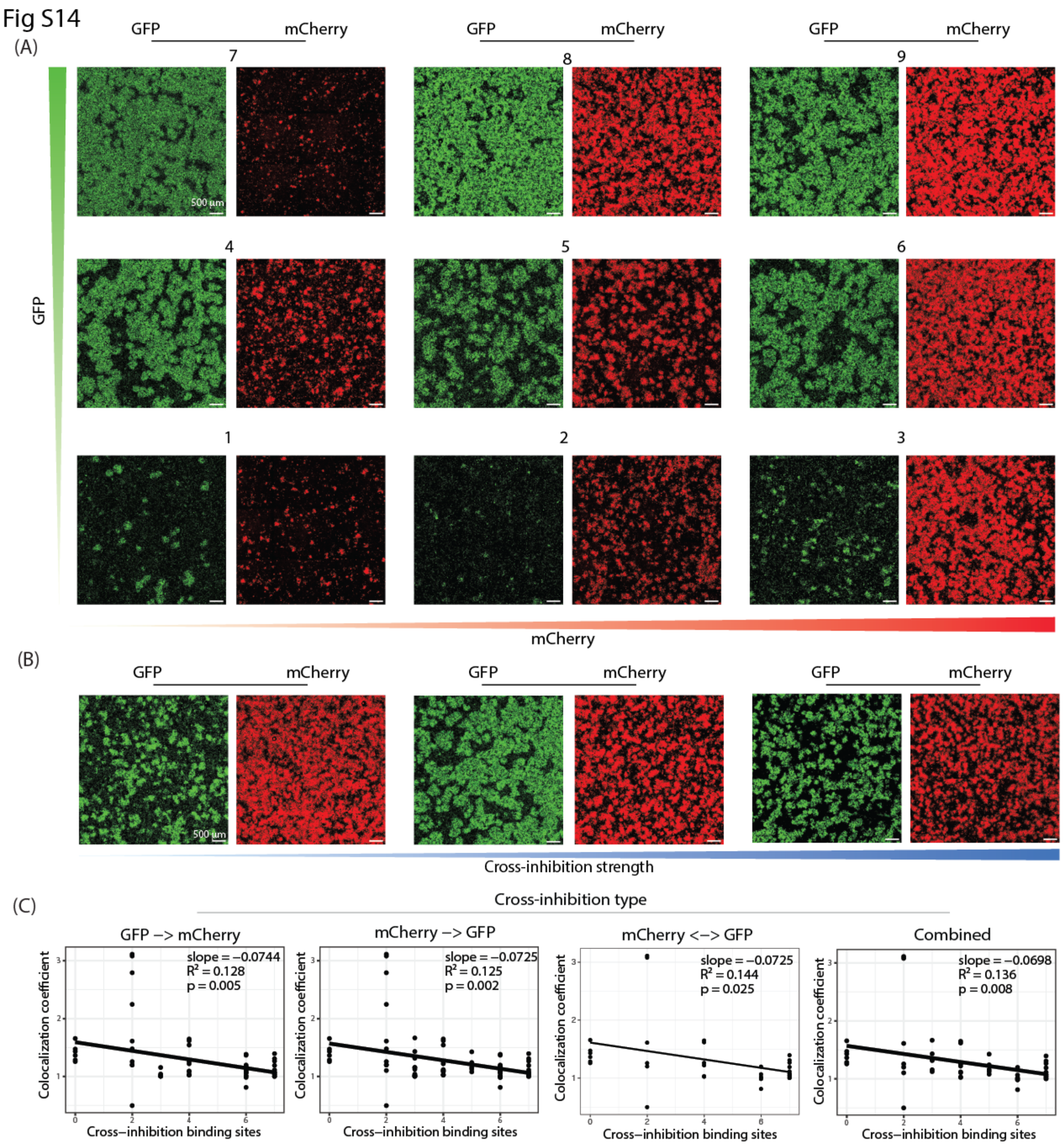

Fig. S14, related to Fig. 5. Characterization of the resulting morphospace from tuneable cross-inhibiting dual-JAPI circuits.

(**A**) Single-channel fluorescence microscope endpoint images at day 4 of nine representative clonal cross-inhibiting dual-JAPI cell lines, corresponding to the merged images shown in Fig. 5H. Initial condition: homogeneous inactivated cell lawn. Scale bar = 500 µm. (**B**) Single-channel fluorescence microscope endpoint images at day 4 of three cross-inhibiting dual-JAPI clonal cell lines with significant negative cross-correlation between GFP and mCherry signals, shown left to right with increasing cross-inhibition strength (indicated by the blue gradient bar below), corresponding to the merged images shown in Fig. 5I. Initial condition: homogeneous inactivated cell lawn. Scale bar = 500 µm. (**C**) Scatter plots with linear regression of the colocalization

bioRxiv preprint doi: https://doi.org/10.64898/2026.05.24.727552; this version posted May 25, 2026. The copyright holder for this preprint (which was not certified by peer review) is the author/funder, who has granted bioRxiv a license to display the preprint in perpetuity. It is made available under a CC-BY 4.0 International license.

coefficient, computed as the ratio of the measured DICE coefficient to the null DICE coefficient, as a function of the number of cross-inhibitory binding sites in the inhibitor promoter, separated by cross-inhibition type. Four sub-panels are shown, from left to right: GFP → mCherry (n = 60 observations from n = 13 genotyped clonal cell lines), mCherry → GFP (n = 51 observations from n = 20 genotyped clonal cell lines), mCherry ↔ GFP (bi-directional, n = 31 observations from n = 7 genotyped clonal cell lines), and combined across all three types (n = 76 observations from n = 25 genotyped clonal cell lines). Each dot is an individual clone; black line is the best-fit linear regression. Slope, R^2, and p-value indicated in the figure for each sub-panel.

bioRxiv preprint doi: https://doi.org/10.64898/2026.05.24.727552; this version posted May 25, 2026. The copyright holder for this preprint (which was not certified by peer review) is the author/funder, who has granted bioRxiv a license to display the preprint in perpetuity. It is made available under a CC-BY 4.0 International license.

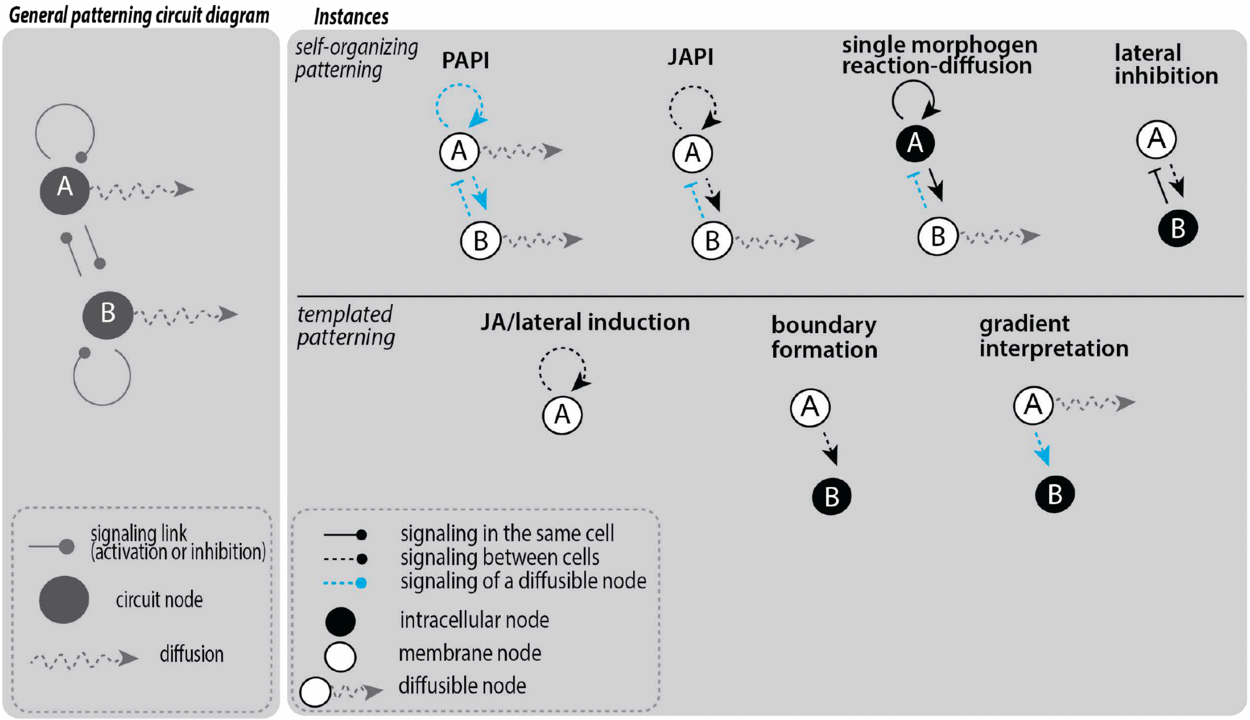

Fig. S15, related to Discussion. A common framework for multicellular patterning circuit architectures

This scheme represents many patterning architectures as variations on a single template. As in standard graph-based representations of patterning circuits, each architecture is built from two nodes (A, B) connected by signaling links. To this we add two independent annotations: one on the nodes, specifying where each species is localized (intracellular, membrane-tethered, or secreted), and one on the links, specifying how each interaction is transmitted (within the same cell, between cells by direct contact, or by diffusion). To define a specific architecture, one specifies which species are present and where they are localized, then which interactions connect them and their type. Because every interaction requires a mode of transport, this view generalizes reaction-diffusion: classical reaction-diffusion (PAPI) is the special case in which all transport occurs by diffusion. The same structure accommodates other reaction-diffusion architectures, such as JAPI, where A is transported by juxtacrine cell-to-cell auto-activation, as well as architectures not classically associated with reaction-diffusion, such as gradient interpretation. Node fill indicates localization: filled, intracellular; open, membrane-tethered; open with a wavy arrow, secreted, where the wavy arrow denotes that the species is itself diffusible. Line style indicates the transport mode of each interaction: solid, within the same cell (cis); dashed black, between cells by direct contact (trans); dashed blue, by diffusion, denoting that a diffusible species acts on a target by diffusing to it. Arrowheads indicate activation and flat bars indicate inhibition. In the general patterning circuit diagram (left), grey nodes and links indicate unspecified localization, transport mode, and sign. On the right, instances are grouped into self-organizing patterning, in which the pattern emerges from the circuit's own dynamics, and templated patterning, in which a pre-existing source instructs the pattern.

Supplementary Notes

bioRxiv preprint doi: https://doi.org/10.64898/2026.05.24.727552; this version posted May 25, 2026. The copyright holder for this preprint (which was not certified by peer review) is the author/funder, who has granted bioRxiv a license to display the preprint in perpetuity. It is made available under a CC-BY 4.0 International license.

Supplementary Note 1
Linear Stability Analysis of JAPI

Model

This note analyzes the linear stability of a juxtacrine-activator paracrine-inhibitor (JAPI) reaction-diffusion system, in which the activator is membrane-tethered and propagates by cell-cell contact, while the inhibitor is paracrine and diffuses. JAPI is contrasted with classical paracrine-activator paracrine-inhibitor (PAPI) systems, the prototypical implementation of local-activation/lateral-inhibition (LALI) reaction-diffusion patterning, in which both species diffuse. We work in units where the lattice spacing is unity, and assume periodic boundary conditions on a one-dimensional lattice (taken either infinite or sufficiently large that finite-size effects are negligible; small finite-lattice corrections are discussed where relevant).

We consider a JAPI system in which the activator is membrane-tethered and propagates through neighbor-mediated relay, while the inhibitor is diffusible. The system is formulated on a one-dimensional lattice of cells indexed by integer j along the lattice (so that j serves simultaneously as cell label and, in unit-spacing units, as spatial coordinate), with the understanding that all cells are geometrically identical and that the lattice is translationally invariant. The juxtacrine interaction kernel is written as $K_{jl} = K(j-l)$, where $K(r)$ is a symmetric, nonnegative, row-normalized function of the cell-to-cell displacement r; its specific form (nearest-neighbor cosine kernel) is given below. In the main text we use the shorthand Ka for the kernel-weighted activator input; here we work with the explicit lattice form $(Ka)_j \equiv \sum_l K_{jl}\, a_l$. Under these assumptions the dynamics take the form

$$\frac{da_j}{dt} = \beta_a\, f\left(\sum_l K_{jl}\, a_l,\ i_j\right) - \mu_a\, a_j \tag{1}$$

$$\frac{di_j}{dt} = \beta_i\, f\left(\sum_l K_{jl}\, a_l,\ i_j\right) - \gamma_i\, i_j + D_i\, \Delta i_j \tag{2}$$

where a_j and i_j denote activator and inhibitor levels in cell j; β_a, β_i are production rates; μ_a, γ_i are degradation rates; and D_i is the inhibitor diffusion coefficient. The symbol Δ denotes the nearest-neighbor discrete Laplacian on the cell index,

$$\Delta i_j \equiv i_{j+1} - 2i_j + i_{j-1}, \tag{3}$$

a finite-difference operator between cells (not a continuum derivative within a cell). The Fourier representation of Δ on the lattice is given in the Fourier Decomposition section below. Both species share the same nonlinear production function f, evaluated at the juxtacrine-weighted activator input $(Ka)_j$ and the local inhibitor level i_j. This coupling structure ensures that the Fourier transform of the kernel, $\hat{K}(q) = \sum_r K(r)\, e^{-iqr}$ (defined precisely in the Fourier Decomposition section), appears in both rows of the linear operator derived below (Turing, 1952; Murray, 2003).

bioRxiv preprint doi: https://doi.org/10.64898/2026.05.24.727552; this version posted May 25, 2026. The copyright holder for this preprint (which was not certified by peer review) is the author/funder, who has granted bioRxiv a license to display the preprint in perpetuity. It is made available under a CC-BY 4.0 International license.

Production function. For the steady state analysis below we take f to be a two-input Hill function representing competitive inhibition,

$$f(a,i) = \frac{(a/k_a)^{n_a}}{1 + (a/k_a)^{n_a} + (i/k_i)^{n_i}} \tag{4}$$

where k_a and k_i are activation and inhibition thresholds and n_a, n_i are Hill coefficients. We take f to be shared between activator and inhibitor because this reflects the experimental implementation (see Fig. 2 in main text), in which both species are driven by a single transcriptional channel: the synNotch receptor activation directly drives expression of both the membrane-tethered activator and the secreted inhibitor through a common promoter. The form of the mode-dependent linear operator extends to the case of distinct production functions $f_a \neq f_i$ with notational changes; the compact determinant identity in the existence proof below is written for the shared-promoter case used here.

Scope of the linear stability analysis. The specific functional form of the production functions determines the location and number of homogeneous steady states, which we analyze for the competitive inhibition case in Supplementary Note 3. The linear stability analysis that follows is, however, agnostic to the specific form of the production functions: it requires only that the steady state (a_0, i_0) exists and that the partial derivatives f_a and f_i evaluated there have the appropriate signs, positive for self-activation and negative for inhibition. The results therefore apply to any reaction-diffusion system with this architecture, not only the competitive inhibition case (Gierer & Meinhardt, 1972; Kondo & Miura, 2010).

Steady State Analysis

Before linearizing, we establish the fixed point structure of the system. Seeking spatially homogeneous solutions $a_j = a$, $i_j = i$, the discrete Laplacian vanishes and the juxtacrine sum reduces to $\sum_l K_{jl}\, a_l = \kappa\, a$, where

$$\kappa \equiv \sum_l K_{jl} \tag{5}$$

is the total juxtacrine coupling weight, independent of j by translational invariance. With the kernel $K_{jl} = K(j-l)$ assumed symmetric ($K(r) = K(-r)$), nonnegative ($K(r) \geq 0$), and row-normalized ($\sum_r K(r) = 1$), the Fourier transform $\hat{K}(q)$ is real, even, and bounded above by $\hat{K}(0) = \kappa = 1$. For unnormalized kernels, the local activator amplification at $q = 0$ becomes $\kappa\, \beta_a\, f_{a0}$, where $f_{a0} \equiv \partial f/\partial a$ evaluated at the activated homogeneous steady state (its explicit form for the competitive Hill function is given in Eq. (15) below), and the conditions derived below should be replaced accordingly with the substitution $\alpha \to \kappa\alpha$. The system reduces to the single-cell ODE:

$$\frac{da}{dt} = \beta_a\, f(\kappa a, i) - \mu_a\, a \tag{6}$$

$$\frac{di}{dt} = \beta_i\, f(\kappa a, i) - \gamma_i\, i \tag{7}$$

Trivial fixed point. The origin $(a,i) = (0,0)$ is always a fixed point. For $n_a > 1$, $f(0,0) = 0$ and $\partial f/\partial a|_{(0,0)} = 0$, so the Jacobian at the origin is diagonal with eigenvalues $-\mu_a$ and $-\gamma_i$, both strictly negative. The off state is therefore linearly stable and does not support Turing instability whenever $n_a > 1$. (For $n_a = 1$ the Jacobian acquires a finite off-diagonal contribution and origin stability becomes parameter-dependent; we do not consider this case here.)

bioRxiv preprint doi: https://doi.org/10.64898/2026.05.24.727552; this version posted May 25, 2026. The copyright holder for this preprint (which was not certified by peer review) is the author/funder, who has granted bioRxiv a license to display the preprint in perpetuity. It is made available under a CC-BY 4.0 International license.

Nontrivial fixed points. Existence of a nontrivial activated fixed point (a_0, i_0) with $a_0 > 0$ depends on both activator and inhibitor parameters in the coupled two-species system. A useful lower bound on the activator production rate required for such a fixed point follows from the reduction of the system to the activation branch alone (called JA, see also main text). A nontrivial fixed point of the JA system satisfies:

$$\beta_a \frac{(\kappa a_0/k_a)^{n_a}}{1 + (\kappa a_0/k_a)^{n_a}} = \mu_a a_0 \tag{8}$$

The onset of bistability in the JA reduction, the parameter value at which a pair of nontrivial fixed points appears, is found by requiring this equation to hold simultaneously with the tangency condition that the production and degradation curves have equal slopes. Solving these two conditions jointly gives the critical activator concentration:

$$a_c = \frac{k_a}{\kappa} (n_a - 1)^{1/n_a} \tag{9}$$

and the critical production rate:

$$\beta_a^c = \frac{\mu_a k_a}{\kappa} \cdot \frac{n_a (n_a - 1)^{1/n_a}}{n_a - 1} \tag{10}$$

Bistability of the JA system requires $n_a > 1$ and $\beta_a > \beta_a^c$. We note that the full bifurcation condition for the coupled (a, i) system is more complex and depends on the inhibitor parameters as well; the JA bistability condition above provides a useful lower bound. A complete analysis of the two-species fixed point structure in dimensionless parameter space, including the dependence on Hill coefficients and the full two-species phase portrait, is provided in Supplementary Note 3.

When the JA bistability condition holds, the one-dimensional JA reduction has three fixed points along the activator axis: the stable off state $(0, 0)$, an unstable threshold state, and a stable activated state. In the full two-species system, the inhibitor tracks the activator at steady state through the ratio $i_0/a_0 = \beta_i \mu_a / (\beta_a \gamma_i)$, which follows from dividing the two steady-state equations.

All subsequent linear stability analysis is performed around the nontrivial activated fixed point (a_0, i_0), whose existence we assume in what follows.

Homogeneous Steady State

We now identify the activated steady state (a_0, i_0) to linearize around. The steady-state values satisfy:

$$\beta_a f(\kappa a_0, i_0) = \mu_a a_0 \tag{11}$$

$$\beta_i f(\kappa a_0, i_0) = \gamma_i i_0 \tag{12}$$

Partial Derivatives of the Production Function

Linearization requires the partial derivatives of f with respect to each argument. Writing $A = (a/k_a)^{n_a}$ and $I = (i/k_i)^{n_i}$, so that $f = A/(1 + A + I)$, direct differentiation gives

$$f_a \equiv \frac{\partial f}{\partial a} = \frac{n_a A (1 + I)}{a (1 + A + I)^2} \tag{13}$$

$$f_i \equiv \frac{\partial f}{\partial i} = -\frac{n_i A I}{i (1 + A + I)^2} \tag{14}$$

bioRxiv preprint doi: https://doi.org/10.64898/2026.05.24.727552; this version posted May 25, 2026. The copyright holder for this preprint (which was not certified by peer review) is the author/funder, who has granted bioRxiv a license to display the preprint in perpetuity. It is made available under a CC-BY 4.0 International license.

Evaluated at the homogeneous state, with $A_0 = (\kappa a_0/k_a)^{n_a}$ and $I_0 = (i_0/k_i)^{n_i}$,

$$f_{a0} = \frac{n_a\, A_0\, (1 + I_0)}{(\kappa a_0)\, (1 + A_0 + I_0)^2} > 0 \tag{15}$$

$$f_{i0} = -\frac{n_i\, A_0\, I_0}{i_0\, (1 + A_0 + I_0)^2} < 0 \tag{16}$$

Note that f_{a0} is evaluated at the juxtacrine input κa_0 rather than a_0 alone, reflecting the fact that the effective activation signal experienced by each cell in the homogeneous state is amplified by the total coupling weight κ.

Linearization

We perturb around the homogeneous state, writing $a_j = a_0 + \delta a_j$ and $i_j = i_0 + \delta i_j$. The perturbed juxtacrine input is

$$\sum_l K_{jl}\, a_l = \kappa\, a_0 + \sum_l K_{jl}\, \delta a_l.$$

Expanding f to first order and canceling steady-state terms using the steady-state equations yields the linearized system

$$\frac{d\,\delta a_j}{dt} = \beta_a\, f_{a0} \sum_l K_{jl}\, \delta a_l + \beta_a\, f_{i0}\, \delta i_j - \mu_a\, \delta a_j \tag{17}$$

$$\frac{d\,\delta i_j}{dt} = \beta_i\, f_{a0} \sum_l K_{jl}\, \delta a_l + \beta_i\, f_{i0}\, \delta i_j - \gamma_i\, \delta i_j + D_i\, \Delta\, \delta i_j \tag{18}$$

The architecture-specific structure is already apparent: the activator couples spatially through the juxtacrine kernel K, while the inhibitor couples through diffusion.

Fourier Decomposition

Cells are indexed by integers j along the 1D lattice, so j serves simultaneously as cell label and (in unit-spacing units) spatial coordinate; both the discrete Laplacian defined above and the Fourier ansatz below rely on this 1D ordered labeling. Extensions to higher-dimensional lattices replace j with a multi-index without changing the structure of the analysis.

We seek normal-mode solutions of the form

$$\delta a_j(t) = \hat{a}\, e^{\lambda t + iqj}, \qquad \delta i_j(t) = \hat{\imath}\, e^{\lambda t + iqj} \tag{19}$$

where $q \in (-\pi, \pi]$ is the lattice wavenumber and λ is the complex growth rate. Under this ansatz the convolution with K diagonalizes,

$$\sum_l K_{jl}\, \delta a_l = \hat{K}(q)\, \delta a_j, \qquad \hat{K}(q) = \sum_r K(r)\, e^{-iqr} \tag{20}$$

At $q = 0$ this reduces to $\hat{K}(0) = \sum_r K(r) = \kappa$, consistent with the homogeneous-state analysis above.

bioRxiv preprint doi: https://doi.org/10.64898/2026.05.24.727552; this version posted May 25, 2026. The copyright holder for this preprint (which was not certified by peer review) is the author/funder, who has granted bioRxiv a license to display the preprint in perpetuity. It is made available under a CC-BY 4.0 International license.

Discrete Laplacian. For nearest-neighbor diffusion on a 1D lattice the discrete Laplacian acting on a Fourier mode gives

$$\Delta\, e^{iqj} = -\Lambda(q)\, e^{iqj}, \qquad \Lambda(q) = 2(1 - \cos q) \geq 0. \tag{21}$$

In the long-wavelength limit $q \to 0$ we have $\Lambda(q) = q^2 + O(q^4)$, recovering the continuum Laplacian eigenvalue and ensuring consistency with reaction-diffusion theory at large spatial scales.

Nearest-neighbor juxtacrine kernel. For a symmetric nearest-neighbor kernel $K(\pm 1) = \frac{1}{2}$, $K(r) = 0$ otherwise, the Fourier transform evaluates to

$$\hat{K}(q) = \cos q. \tag{22}$$

This is not a delta function (whose transform would be flat); rather it is a finite-range kernel whose Fourier transform is positive at low q (coherent amplification of long-wavelength modes) and becomes negative near $q = \pi$ (suppression of checkerboard modes). The sign change at high wavenumber is a qualitative feature of discrete juxtacrine coupling that has no analog in diffusive activator spread, though at low wavenumbers the cosine kernel admits an effective-diffusion rewriting (analyzed in the comparison section below). The nearest-neighbor kernel is the natural minimal model for juxtacrine signaling in which cells interact only with immediate neighbors; more extended or graded kernels (e.g. a Gaussian $K(x) \propto e^{-x^2/2s^2}$) can be introduced if a continuously tunable interaction range is desired, but introduce an additional phenomenological length scale not analyzed here.

Eigenvalue problem. Substituting the Laplacian and kernel transforms into the linearized dynamics, the system reduces for each mode q to the 2×2 eigenvalue problem

$$\lambda \begin{pmatrix} \hat{a} \\ \hat{\imath} \end{pmatrix} = \mathbf{A}(q) \begin{pmatrix} \hat{a} \\ \hat{\imath} \end{pmatrix} \tag{23}$$

with linear operator

$$\mathbf{A}(q) = \begin{pmatrix} -\mu_a + \beta_a\, f_{a0}\, \hat{K}(q) & \beta_a\, f_{i0} \\ \beta_i\, f_{a0}\, \hat{K}(q) & -\gamma_i + \beta_i\, f_{i0} - D_i\, \Lambda(q) \end{pmatrix} \tag{24}$$

This is the central linear operator for JAPI.

Dispersion Relation

The eigenvalues of $\mathbf{A}(q)$ are found from the characteristic equation

$$\lambda^2 - \tau(q)\, \lambda + \Delta(q) = 0 \tag{25}$$

where the trace and determinant are

$$\tau(q) = \left(-\mu_a + \beta_a\, f_{a0}\, \hat{K}(q)\right) + \left(-\gamma_i + \beta_i\, f_{i0} - D_i\, \Lambda(q)\right) \tag{26}$$

$$\Delta(q) = \left(-\mu_a + \beta_a\, f_{a0}\, \hat{K}(q)\right)\left(-\gamma_i + \beta_i\, f_{i0} - D_i\, \Lambda(q)\right) - \beta_a\, f_{i0} \cdot \beta_i\, f_{a0}\, \hat{K}(q) \tag{27}$$

The two eigenvalues are

$$\lambda_\pm(q) = \tfrac{1}{2}\left[\tau(q) \pm \sqrt{\tau(q)^2 - 4\Delta(q)}\right] \tag{28}$$

and the relevant dispersion relation is $\lambda_{\max}(q) = \max\{\mathrm{Re}(\lambda_+), \mathrm{Re}(\lambda_-)\}$. A patterned state arises when $\lambda_{\max}(q) > 0$ for at least one mode $q \neq 0$ while the $q = 0$ mode remains stable, and the dominant pattern wavelength at onset is set by the mode q^* that maximizes $\lambda_{\max}(q)$.

bioRxiv preprint doi: https://doi.org/10.64898/2026.05.24.727552; this version posted May 25, 2026. The copyright holder for this preprint (which was not certified by peer review) is the author/funder, who has granted bioRxiv a license to display the preprint in perpetuity. It is made available under a CC-BY 4.0 International license.

Stability Conditions

Homogeneous stability. The $q = 0$ mode corresponds to spatially uniform perturbations. Since $\Lambda(0) = 0$ and $\hat{K}(0) = \kappa$, stability requires

$$\tau(0) < 0 \quad \text{and} \quad \Delta(0) > 0. \tag{29}$$

These are the standard Routh–Hurwitz conditions for the local reaction system, ensuring that the homogeneous steady state is stable to uniform perturbations.

Spatial instability. A patterned state emerges when the homogeneous conditions hold yet $\lambda_{\max}(q) > 0$ for some $q \neq 0$. Under the trace-negative conditions verified in the next subsection, this is equivalent to $\Delta(q)$ becoming negative at some nonzero wavenumber, here controlled by the interplay between $\hat{K}(q)$ and $\Lambda(q)$ rather than by two diffusion coefficients. The existence of parameter regimes where this occurs is proven in the next subsection.

Existence of Turing Instabilities in JAPI

We show that the JAPI operator $\mathbf{A}(q)$ derived above admits finite-wavenumber instabilities of the homogeneous on state under the standard local activator–inhibitor prerequisites, establishing that the Turing regime is accessible to the JAPI architecture. The closed-form threshold, mode selection, and unstable band are the object of future theoretical work; here we establish existence.

Setup and prerequisites. Introduce the abbreviations

$$\alpha \equiv \beta_a\, f_{a0} > 0, \qquad \nu \equiv -\beta_i\, f_{i0} > 0, \tag{30}$$

where positivity follows from $f_{a0} > 0$ and $f_{i0} < 0$ at the activated steady state. *Sign convention.* The minus sign in the definition of ν is chosen so that ν is positive; with this convention $\beta_i f_{i0} = -\nu$, so the (2,2) entry of $\mathbf{A}(q)$ reads $-\gamma_i - \nu - D_i\Lambda(q)$. All expressions below (in particular the determinant identity in Eq. (32)) follow this convention. An alternative convention in which ν is defined as $\beta_i f_{i0}$ (negative-valued) would give equivalent results with sign flips wherever ν appears; e.g., the leading term of Eq. (32) would read $\mu_a(\gamma_i - \nu)$ rather than $\mu_a(\gamma_i + \nu)$, with the same numerical value because of the sign flip in ν. The local prerequisites for a Turing-type instability of the on state are:

(i) $\alpha > \mu_a$ (local activator self-amplification at $q = 0$)

(ii) $\alpha - \mu_a - \gamma_i - \nu < 0$ (Routh–Hurwitz trace condition at $q = 0$)

(iii) $\mu_a\nu > \gamma_i(\alpha - \mu_a)$ (Routh–Hurwitz determinant condition at $q = 0$)

Conditions (ii) and (iii) together ensure homogeneous stability of the on state.

We additionally assume that the lattice kernel and Laplacian eigenvalue satisfy the normalization properties

$$\hat{K}(0) = 1, \qquad \Lambda(0) = 0, \qquad \Lambda(q) > 0 \text{ for } q \neq 0, \qquad \hat{K}(q) \leq 1, \tag{31}$$

and that the lattice spectrum contains at least one allowed nonzero mode in a sufficiently small neighborhood of $q = 0$ where the continuity arguments below apply. This is automatic for an infinite lattice or for a sufficiently large finite lattice, but should be checked mode-by-mode on small finite lattices. For the cosine kernel $\hat{K}(q) = \cos q$, this holds in the continuous-q or sufficiently large finite-lattice limit whenever $\alpha > \mu_a$; on small finite lattices the allowed modes must be checked explicitly.

bioRxiv preprint doi: https://doi.org/10.64898/2026.05.24.727552; this version posted May 25, 2026. The copyright holder for this preprint (which was not certified by peer review) is the author/funder, who has granted bioRxiv a license to display the preprint in perpetuity. It is made available under a CC-BY 4.0 International license.

Determinant structure. Direct expansion of $\det \mathbf{A}(q)$ using Eq. (24), after using $\beta_a f_{i0} \cdot \beta_i f_{a0} = -\alpha\nu$, yields

$$\Delta(q) \;=\; \mu_a(\gamma_i + \nu) \;-\; \alpha\gamma_i \hat{K}(q) \;+\; D_i \Lambda(q)\big(\mu_a - \alpha\hat{K}(q)\big). \tag{32}$$

The right-hand side separates into a q-dependent local part, $\mu_a(\gamma_i + \nu) - \alpha\gamma_i\hat{K}(q)$, and a spatial-coupling part $D_i \Lambda(q)(\mu_a - \alpha\hat{K}(q))$ that is linear in D_i. The coefficient of D_i is determined by the sign of $\mu_a - \alpha\hat{K}(q)$: for modes where $\alpha\hat{K}(q) > \mu_a$, the coefficient is negative, and $\Delta(q)$ decreases linearly as D_i increases.

All modes are stable at $D_i = 0$. Before showing that finite D_i can destabilize a nonzero mode, we verify that no spatial instability exists in the absence of inhibitor diffusion. At $D_i = 0$,

$$\Delta(q, 0) = \mu_a(\gamma_i + \nu) - \alpha\gamma_i \hat{K}(q). \tag{33}$$

Using $\hat{K}(q) \le 1$,

$$\Delta(q, 0) \;\ge\; \mu_a(\gamma_i + \nu) - \alpha\gamma_i \;=\; \mu_a\nu - \gamma_i(\alpha - \mu_a) \;>\; 0 \tag{34}$$

by condition (iii). The trace is

$$\operatorname{tr} \mathbf{A}(q, 0) = -\mu_a + \alpha\hat{K}(q) - \gamma_i - \nu \;\le\; -\mu_a + \alpha - \gamma_i - \nu \;<\; 0 \tag{35}$$

by condition (ii). All modes are therefore linearly stable at $D_i = 0$, regardless of wavenumber. Spatial instability in JAPI is consequently *diffusion-driven*: it requires $D_i > 0$ and emerges as D_i increases beyond a finite threshold, in the standard Turing sense.

Existence of a destabilizing finite-wavenumber mode. By continuity of $\hat{K}(q)$ and condition (i), there exists $q^* \neq 0$ sufficiently close to $q = 0$ such that both

$$\alpha \hat{K}(q^*) > \mu_a \tag{36}$$

$$N(q^*) \;\equiv\; \mu_a(\gamma_i + \nu) - \alpha\gamma_i \hat{K}(q^*) > 0 \tag{37}$$

hold. The first follows from $\alpha \hat{K}(0) = \alpha > \mu_a$, and the second follows from $N(0) = \mu_a\nu - \gamma_i(\alpha - \mu_a) > 0$ (condition iii), both by continuity.

For such a q^*, write

$$\Delta(q^*) \;=\; N(q^*) \;-\; D_i M(q^*), \tag{38}$$

where $M(q^*) \equiv \Lambda(q^*)\big(\alpha\hat{K}(q^*) - \mu_a\big) > 0$. The determinant $\Delta(q^*)$ is therefore a strictly decreasing linear function of D_i with positive intercept $N(q^*)$ and positive slope magnitude $M(q^*)$. It vanishes at the finite positive threshold

$$D_{\text{crit}}(q^*) \;=\; \frac{N(q^*)}{M(q^*)} \;>\; 0, \tag{39}$$

and becomes negative for all $D_i > D_{\text{crit}}(q^*)$. At this mode, the JAPI operator therefore acquires a positive-real-part eigenvalue, while the homogeneous mode remains stable because $q = 0$ is unaffected by D_i.

Existence statement. Under conditions (i)–(iii) and the lattice spectral assumptions stated above, the JAPI operator $\mathbf{A}(q)$ admits at least one wavenumber $q^* \neq 0$ at which the homogeneous on state is destabilized by sufficiently strong inhibitor diffusion, while the homogeneous mode remains stable. This is the defining signature of a Turing-type instability.

Because the off state $(0, 0)$ remains linearly stable for $n_a > 1$ (see Trivial fixed point analysis above), this argument establishes linear instability of the activated homogeneous branch only, not global

bioRxiv preprint doi: https://doi.org/10.64898/2026.05.24.727552; this version posted May 25, 2026. The copyright holder for this preprint (which was not certified by peer review) is the author/funder, who has granted bioRxiv a license to display the preprint in perpetuity. It is made available under a CC-BY 4.0 International license.

convergence of the nonlinear system to a patterned state. Nonlinear pattern selection, saturation, and the possibility of basins of attraction containing the off state require numerical simulation or weakly nonlinear analysis, and are not the object of this note.

The JAPI architecture therefore admits Turing instabilities under the same type of local activator–inhibitor prerequisites familiar from classical PAPI, a comparison developed in detail in the next subsection, with the destabilizing spatial threshold provided by D_i alone rather than by a ratio of two diffusion coefficients. The precise threshold

$$D_T^{\mathrm{JAPI}} = \inf_{\substack{q \neq 0 \\ \alpha \hat{K}(q) > \mu_a}} D_{\mathrm{crit}}(q) \tag{40}$$

(replaced by a minimum over allowed lattice modes $q_m = 2\pi m/N$ on a finite periodic lattice of N cells) depends on the lattice geometry through $\hat{K}(q)$ and $\Lambda(q)$, and on the lattice spectrum on finite lattices. For the 1D nearest-neighbor cosine kernel in the continuous-q limit, the infimum is attained in the interior of the allowed band under the stated conditions and admits a direct closed-form expression by minimization of $D_{\mathrm{crit}}(q)$ over q. We do not pursue this in detail here; closed-form analysis of D_T^{JAPI} for general kernels, finite-lattice corrections, higher-dimensional geometries, and the relationship to the classical PAPI threshold, are the object of future theoretical work.

Worked example. The following parameter set is presented as a *local-Jacobian* demonstration of existence: it satisfies conditions (i)–(iii) and the kernel assumptions, and so admits a Turing instability of the operator $\mathbf{A}(q)$. We do not claim it corresponds to a particular set of Hill-function parameters $(\beta_a, \beta_i, k_a, k_i, n_a, n_i)$ satisfying the homogeneous steady-state equations; for the competitive Hill production function, the steady-state constraint imposes $\alpha/\mu_a < n_a$ and $\nu/\gamma_i < n_i$, so realizing the example below from the Hill model would require $n_a > 5$ and $n_i > 10$, which are higher than the experimentally measured Hill coefficients in the implementation analyzed in the main text. A Hill-realizable parameter set in the irregular regime relevant to the experimental implementation is analyzed in future theoretical work; the example here serves only to exhibit a Jacobian-level parameter set in which the existence proof applies.

Take

$$\alpha = 5, \quad \mu_a = 1, \quad \gamma_i = 0.5, \quad \nu = 5, \tag{41}$$

with the 1D nearest-neighbor kernel $\hat{K}(q) = \cos q$. Verification of (i)–(iii):

(i) $\alpha - \mu_a = 4 > 0$ ✓

(ii) $\alpha - \mu_a - \gamma_i - \nu = -1.5 < 0$ ✓

(iii) $\mu_a \nu - \gamma_i(\alpha - \mu_a) = 3 > 0$ ✓

Pick q^* with $\cos q^* = 0.8$. Then $\alpha \hat{K}(q^*) = 4 > \mu_a$, $\Lambda(q^*) = 0.4$, and

$$\begin{aligned} N(q^*) &= 1 \cdot 5.5 - 5 \cdot 0.5 \cdot 0.8 = 3.5 > 0 \\ M(q^*) &= 0.4 \cdot (4 - 1) = 1.2 \\ D_{\mathrm{crit}}(q^*) &= 3.5/1.2 \approx 2.92. \end{aligned}$$

For any $D_i > 2.92$, the mode q^* satisfies $\Delta(q^*) < 0$. At $q = 0$, both Routh–Hurwitz conditions hold regardless of D_i: $\tau(0) = -1.5 < 0$ and $\Delta(0) = 3 > 0$, so the homogeneous mode remains stable. The system is therefore in a Turing regime for $D_i > 2.92$ at this parameter set. The chosen q^* (with $\cos q^* = 0.8$) is illustrative; direct numerical minimization of $D_{\mathrm{crit}}(q)$ over the allowed band gives

bioRxiv preprint doi: https://doi.org/10.64898/2026.05.24.727552; this version posted May 25, 2026. The copyright holder for this preprint (which was not certified by peer review) is the author/funder, who has granted bioRxiv a license to display the preprint in perpetuity. It is made available under a CC-BY 4.0 International license.

the true onset $D_T \approx 2.46$ for this parameter set, at the optimal mode $\cos q_T \approx 0.65$, $q_T \approx 0.86$ rad (wavelength ≈ 7.3 cells).

Comment on the geometric threshold. For the 1D nearest-neighbor cosine kernel, the expression

$$D_{\text{geom}} = \frac{\alpha \gamma_i}{2(\alpha - \mu_a)} = 0.3125 \tag{42}$$

for the parameters above, obtained by requiring that the determinant minimum lie within the physical interval of accessible wavenumbers, has appeared in informal analyses as a candidate threshold. D_{geom} is strictly necessary for finite-q instability (it gives the lower bound on D_i for the minimum of $\Delta(c)$ over $c = \cos q$ to fall in the physical band) but is *not* the threshold itself: $D_T^{\text{JAPI}} > D_{\text{geom}}$ in general. A direct numerical minimization of $D_{\text{crit}}(q)$ gives $D_T \approx 2.46$ for the parameter set above. A closed-form expression for D_T^{JAPI}, including for general kernels, is developed in future theoretical work.

Structure of the Linear Operator: Comparison with Classical LALI

We now compare the JAPI linear operator derived above with the corresponding operator for a classical paracrine activator–paracrine inhibitor (PAPI) system. The comparison clarifies which features are shared between the two architectures and which are specific to JAPI.

For PAPI, an identical linearization procedure yields

$$\mathbf{A}_{\text{PAPI}}(q) = \begin{pmatrix} -\mu_a + \beta_a f_{a0} - D_a \Lambda(q) & \beta_a f_{i0} \\ \beta_i f_{a0} & -\gamma_i + \beta_i f_{i0} - D_i \Lambda(q) \end{pmatrix} \tag{43}$$

where D_a is the activator diffusion coefficient and $\Lambda(q)$ is the same discrete Laplacian eigenvalue as in the JAPI case. A note on notation: f_{a0} in the PAPI operator is evaluated at the homogeneous state a_0 (no juxtacrine input), and so is formally distinct from f_{a0} in JAPI which is evaluated at the juxtacrine input κa_0. For the symmetric nearest-neighbor kernel $K(\pm 1) = 1/2$ used here, $\kappa = 1$ and the two evaluations coincide; the structural comparison below holds in the general case as well.

Placing the two operators side by side:

$$\mathbf{A}_{\text{JAPI}}(q) = \begin{pmatrix} -\mu_a + \beta_a f_{a0} \hat{K}(q) & \beta_a f_{i0} \\ \beta_i f_{a0} \hat{K}(q) & -\gamma_i + \beta_i f_{i0} - D_i \Lambda(q) \end{pmatrix} \tag{44}$$

The two operators are identical except in the entries involving the activator's spatial coupling. Specifically:

- **Entry (1,1).** In PAPI, the activator self-term carries $-D_a \Lambda(q)$, a diffusive penalty whose q-dependent shape $\Lambda(q)$ is fixed by the lattice geometry and whose amplitude D_a is an independent biophysical parameter, tunable without affecting the local reaction kinetics. In JAPI, this is replaced by $\beta_a f_{a0} \hat{K}(q)$, where the q-dependent shape $\hat{K}(q)$ is again fixed by the lattice geometry but the amplitude is set by $\beta_a f_{a0}$, the same coupling that determines the local self-amplification at $q = 0$. The kernel shapes are fixed by lattice geometry in both architectures; what differs is whether the amplitude is independent of the reaction kinetics (D_a in PAPI, yes) or entangled with them ($\beta_a f_{a0}$ in JAPI, no).
- **Entry (2,1).** In PAPI, the inhibitor cross-term $\beta_i f_{a0}$ carries no $\hat{K}(q)$ factor because inhibitor production responds to the local activator perturbation rather than to a juxtacrine-weighted

bioRxiv preprint doi: https://doi.org/10.64898/2026.05.24.727552; this version posted May 25, 2026. The copyright holder for this preprint (which was not certified by peer review) is the author/funder, who has granted bioRxiv a license to display the preprint in perpetuity. It is made available under a CC-BY 4.0 International license.

activator input. In JAPI, this entry carries $\beta_i\, f_{a0}\, \hat{K}(q)$ because the inhibitor is produced in response to the juxtacrine activator input, which is itself mode-dependent. The kernel therefore modulates not only the activator self-term but also the inhibitor cross-coupling.

- **Entries (2,2) and (1,2).** Identical in both systems. The inhibitor diffusion term $-D_i\Lambda(q)$ and the inhibitory feedback $\beta_a\, f_{i0}$ are unchanged.

The key consequence is that D_a drops out of the JAPI patterning problem entirely (Marcon et al., 2016). The instability mechanism is qualitatively preserved: both architectures combine local activator self-amplification with inhibitor-mediated long-range suppression. However, JAPI implements the activator-side spatial structure through the juxtacrine kernel rather than through an independently tunable activator diffusion coefficient.

For convenience in the discussion that follows, we denote the diagonal entries of the JAPI operator as $P(q) \equiv -\mu_a + \beta_a\, f_{a0}\, \hat{K}(q)$ and $Q(q) \equiv -\gamma_i + \beta_i\, f_{i0} - D_i\, \Lambda(q)$ (activator and inhibitor self-couplings, respectively). The inhibitor self-coupling $Q(q)$ is identical in both architectures and always negative: $f_{i0} < 0$ ensures the inhibitor self-loop is stabilizing, and $-D_i\Lambda(q)$ makes $Q(q)$ more negative at higher q. The instability therefore requires $P(q) > 0$ at some nonzero wavenumber, meaning $\beta_a\, f_{a0}\, \hat{K}(q) > \mu_a$ at those modes.

What differs between the two systems is the parametric structure of $P(q)$. In PAPI, $P_{\text{PAPI}}(q) = -\mu_a + \beta_a\, f_{a0} - D_a\Lambda(q)$, and the amplitude on the spatial term is D_a, an independent molecular parameter that can be tuned without affecting the local reaction kinetics: decreasing D_a keeps $P_{\text{PAPI}}(q)$ positive over a wider range of wavenumbers and widens the unstable band. In JAPI, $P_{\text{JAPI}}(q) = -\mu_a + \beta_a\, f_{a0}\, \hat{K}(q)$, and the amplitude on the spatial term is $\beta_a\, f_{a0}$, which is not independent of the reaction kinetics: the same coupling sets both the $q = 0$ self-amplification (via $\beta_a\, f_{a0}$ directly) and the q-dependent activator coupling (via $\beta_a\, f_{a0}\, \hat{K}(q)$). This entanglement is the precise sense in which JAPI is a more constrained system: the qualitative instability mechanism is the same, but the independent tuning of activator spatial spread through a parameter decoupled from the reaction kinetics, which D_a provides in PAPI, has been removed by construction.

For the nearest-neighbor kernel, $\hat{K}(q) = \cos q = 1 - \Lambda(q)/2$. Substituting into the JAPI activator self-coupling gives

$$P_{\text{JAPI}}(q) = -\mu_a + \alpha\, \hat{K}(q) = (-\mu_a + \alpha) - \frac{\alpha}{2}\, \Lambda(q), \tag{45}$$

which has exactly the same q-dependence as a PAPI activator self-coupling with an effective activator diffusion coefficient $D_a^{\text{eff}} = \alpha/2$. In this sense, the JAPI (1,1) entry is diffusion-like in the nearest-neighbor case, but with the effective diffusion amplitude slaved to the local activator gain $\alpha = \beta_a\, f_{a0}$ rather than tunable independently. The genuinely architecture-specific structural distinction is therefore not the form of the (1,1) entry, but the kernel filtering of the (2,1) off-diagonal entry: in PAPI the activator-to-inhibitor coupling carries no q-dependence, while in JAPI it carries a $\hat{K}(q)$ factor inherited from the juxtacrine activator input. For more extended kernels, the (1,1) entry can also deviate from the standard diffusive q^2 form at intermediate and high wavenumbers; in the 1D nearest-neighbor case analyzed here, the deviation is small at low q but the sign change of $\cos q$ near $q = \pi$ remains a qualitative difference from Fickian diffusion. Closed-form analysis of selected wavenumber, instability bandwidth, and parameter space geometry that follow from these distinctions, including the consequences of the (2,1) entry's kernel filtering, are the object of future theoretical work.

bioRxiv preprint doi: https://doi.org/10.64898/2026.05.24.727552; this version posted May 25, 2026. The copyright holder for this preprint (which was not certified by peer review) is the author/funder, who has granted bioRxiv a license to display the preprint in perpetuity. It is made available under a CC-BY 4.0 International license.

Summary

Linear stability analysis of JAPI yields a 2×2 mode-dependent growth operator $\mathbf{A}(q)$ in which juxtacrine relay enters through the Fourier transform of the neighbor kernel $\hat{K}(q)$, while inhibitor diffusion contributes the stabilizing penalty $-D_i\Lambda(q)$. Under the standard local activator–inhibitor prerequisites for Turing patterning, we prove that the JAPI operator admits finite-wavenumber instabilities, establishing that the Turing regime is accessible to the JAPI architecture. The result is a sufficient condition stated at the Jacobian level: it establishes that the JAPI architecture admits Turing-type instability whenever the local activator–inhibitor prerequisites are met at some activated steady state, independent of whether a given Hill parameterization realizes those prerequisites. Direct comparison with the classical PAPI operator shows that the two systems differ in exactly two entries, both involving the activator spatial coupling, while sharing identical inhibitor diffusion, inhibitory feedback, and local reaction structure.

The instability mechanism is qualitatively preserved in JAPI: Turing-type patterning requires local activator self-amplification ($P(q) > 0$ at some nonzero wavenumber) combined with long-range inhibitory suppression ($Q(q) < 0$ driven by $D_i\Lambda(q)$). What JAPI loses relative to PAPI is D_a as an independent tuning parameter for the width of the unstable band. The activator spatial term is instead fixed by kernel geometry and circuit gain $\beta_a f_{a0}$, which are entangled with the rest of the reaction network. The closed-form threshold expression for the JAPI Turing onset, its precise relationship to the classical PAPI threshold, and the structure of the unstable band are the object of future theoretical work.

References

1. Turing, A. M. (1952). The chemical basis of morphogenesis. *Philosophical Transactions of the Royal Society of London. Series B, Biological Sciences*, 237(641), 37–72.

2. Gierer, A. & Meinhardt, H. (1972). A theory of biological pattern formation. *Kybernetik*, 12(1), 30–39.

3. Murray, J. D. (2003). *Mathematical Biology II: Spatial Models and Biomedical Applications* (3rd ed.). Springer-Verlag, New York.

4. Kondo, S. & Miura, T. (2010). Reaction-diffusion model as a framework for understanding biological pattern formation. *Science*, 329(5999), 1616–1620.

5. Marcon, L., Diego, X., Sharpe, J. & Müller, P. (2016). High-throughput mathematical analysis identifies Turing networks for patterning with equally diffusing signals. *eLife*, 5, e14022.

bioRxiv preprint doi: https://doi.org/10.64898/2026.05.24.727552; this version posted May 25, 2026. The copyright holder for this preprint (which was not certified by peer review) is the author/funder, who has granted bioRxiv a license to display the preprint in perpetuity. It is made available under a CC-BY 4.0 International license.

Supplementary Note 2

Physical Interpretation of the Juxtacrine Activator Velocity Scale

Supplementary Note 1 establishes that the activator diffusion coefficient D_a does not appear in the JAPI patterning problem, and proves the existence of Turing-type finite-wavenumber instabilities under the standard local prerequisites. The Note 1 analysis identifies the conditions under which spatially patterned states can arise. The present note addresses a complementary question: how rapidly an activated domain expands through the tissue before inhibitor-mediated arrest. To this end, we develop the physical interpretation of what governs the activator's spatial propagation in JAPI: an effective propagation velocity v_a characterizing the cell-to-cell relay of the membrane-tethered activator. We derive a minimal threshold-crossing estimate for v_a in an inhibitor-suppressed limit; a full traveling-wave analysis incorporating the coupled neighbor dynamics, lattice-discreteness effects, and inhibitor-mediated corrections is the subject of future theoretical work.

Setup

For reference, the full PAPI and JAPI dynamics take the forms:

$$\text{PAPI:}\quad \frac{\partial a}{\partial t} = D_a \nabla^2 a + \beta_a f(a,i) - \mu_a a \tag{1}$$

$$\frac{\partial i}{\partial t} = D_i \nabla^2 i + \beta_i f(a,i) - \gamma_i i \tag{2}$$

$$\text{JAPI:}\quad \frac{da_j}{dt} = \beta_a\, f\left(\sum_l K_{jl}\, a_l,\ i_j\right) - \mu_a\, a_j \tag{3}$$

$$\frac{di_j}{dt} = \beta_i\, f\left(\sum_l K_{jl}\, a_l,\ i_j\right) - \gamma_i\, i_j + D_i\, \Delta i_j \tag{4}$$

The PAPI activator equation contains the diffusion term $D_a \nabla^2 a$; the JAPI activator equation replaces it with the discrete kernel sum over neighboring cells. The PAPI equations are written in continuum, appropriate for the diffusive regime; the JAPI equations are written on a lattice, reflecting the cell-by-cell architecture of the relay (Murray, 2003). The juxtacrine kernel K_{jl} is taken to be nonnegative, symmetric, row-normalized, and self-input free ($K_{jj} = 0$), as stated in Supplementary Note 1.

The full coupled four-equation system is analyzed in Supplementary Note 1 (linearization and stability) and Supplementary Note 4 (arrest dynamics). For the velocity-scale analysis that follows, we work in an inhibitor-suppressed limit appropriate to the early expansion phase of an activated domain: before significant inhibitor accumulation, the inhibitor argument of f contributes negligibly to activator production. Operationally, we set $f(\cdot, i_j) \approx f(\cdot, 0)$, so the activator equation reduces to a one-species front-propagation problem driven by activator self-amplification and degradation alone. This limit also describes exactly the transceiver configuration where the inhibitor branch is genetically absent. The circuit parameters governing v_a in this limit are derived and discussed

bioRxiv preprint doi: https://doi.org/10.64898/2026.05.24.727552; this version posted May 25, 2026. The copyright holder for this preprint (which was not certified by peer review) is the author/funder, who has granted bioRxiv a license to display the preprint in perpetuity. It is made available under a CC-BY 4.0 International license.

below; the inhibitor parameters re-enter the dynamics when the system transitions from expansion to arrest, which is the subject of Supplementary Note 4.

Physical picture of juxtacrine relay

In PAPI, the activator spreads through a tissue by diffusion. Its reach at any position is characterized by the diffusion length $\lambda_a = \sqrt{D_a/\mu_a}$, which captures the steady-state balance between diffusion and degradation in the linearized reaction-diffusion equation.

In JAPI, the membrane-tethered activator does not diffuse. Instead, an activated cell presents activator ligand to its immediate neighbors through cell-cell contact. When a neighbor receives a juxtacrine signal, it begins producing its own activator ligand, which it presents to its own neighbors. The sensitivity of a cell to its kernel-weighted input $s_j = \sum_l K_{jl}\, a_l$ is set by k_a, the input concentration at which production is half-maximal. The activator "spreads" through this relay, advancing one cell at a time as activator accumulates and drives production in successive neighbors.

The rate of this relay defines an effective propagation velocity v_a for the activation front. Rather than a length scale set by diffusion, JAPI has a velocity set by relay kinetics. Note that, unlike paracrine diffusion, the relay in JAPI is intrinsically discrete: the cell is the unit of advance, and the front advances in integer cell-diameter steps rather than as a continuous spatial process. This discrete relay mode is a lattice analog of front propagation in reaction-diffusion systems (Kolmogorov et al., 1937). The precise classification (Fisher-KPP, Zeldovich, or other type), and how it differs from the corresponding classical PAPI fronts given JAPI's lattice discreteness and the absence of an independent activator diffusion coefficient, is the subject of future theoretical work.

Threshold-crossing estimate

To make v_a concrete, we consider a single relay step: a previously inactive cell j exposed to juxtacrine signal from already-activated neighbors. Writing the kernel-weighted incoming signal to cell j as $s_j(t) = \sum_l K_{jl}\, a_l(t)$, the internal activator $a_j(t)$ evolves (in the inhibitor-suppressed limit) as

$$\frac{da_j}{dt} = \beta_a\, F(s_j) - \mu_a\, a_j, \tag{5}$$

where $F(s) = s^{n_a}/(k_a^{n_a} + s^{n_a})$ is the activation-side Hill function and k_a is the half-maximal input concentration of the receiver.

Two distinct thresholds appear naturally in a relay step, because the input to a downstream cell is kernel-weighted rather than equal to the activator level of the upstream cell. Cell $j+1$ activates when its input $s_{j+1} = \sum_l K_{j+1,l}\, a_l$ crosses k_a. If the only contribution to s_{j+1} comes from cell j (worst case, no contribution from the other side), then cell j must accumulate an activator level $a_j = k_a/K_{j+1,j}$ before s_{j+1} reaches k_a; this elevated level is the *output threshold* θ_a. More generally, $\theta_a \sim k_a/\sum_l K_{lj}$, with the precise factor depending on lattice geometry and front orientation. For the symmetric nearest-neighbor coupling used here ($K(\pm 1) = 1/2$), the two thresholds differ by an $O(1)$ geometric prefactor, and we identify a_j's threshold-crossing condition with $a_j = k_a$ for the one-step estimate that follows. (For identical cells with a delta-function kernel $K_{j+1,j} = 1$ the two thresholds coincide; the distinction arises whenever the kernel distributes the activator signal across multiple neighbors.) A self-consistent treatment distinguishing input and output thresholds enters the full traveling-wave analysis and is deferred to future theoretical work.

bioRxiv preprint doi: https://doi.org/10.64898/2026.05.24.727552; this version posted May 25, 2026. The copyright holder for this preprint (which was not certified by peer review) is the author/funder, who has granted bioRxiv a license to display the preprint in perpetuity. It is made available under a CC-BY 4.0 International license.

Quasi-static approximation. We assume that during the threshold-crossing event of cell j, the input $s_j(t)$ is approximately constant, with the activated neighbors at their quasi-steady-state activator levels on the timescale of j's activation. Concretely, $s_j^* = \sum_{l \in A} K_{jl}\, a_l^*$, where A is the set of already-active neighbors and $a_l^* \approx \beta_a/\mu_a$ in the well-saturated limit ($F(s_l^*) \to 1$). This is a leading-order approximation; a self-consistent treatment of the leading-edge cells, where upstream activator levels and their inputs evolve together, is part of the full traveling-wave analysis deferred to future theoretical work.

Under this approximation, define $\beta_{\text{eff}} \equiv \beta_a\, F(s_j^*)$, where s_j^* is the (constant) input to cell j. Starting from $a_j(0) = 0$, the cell ODE has the closed-form solution

$$a_j(t) = \frac{\beta_{\text{eff}}}{\mu_a} \left(1 - e^{-\mu_a t}\right). \tag{6}$$

The threshold-crossing time t_{th} at which $a_j(t_{\text{th}}) = k_a$ is

$$t_{\text{th}} = -\frac{1}{\mu_a} \ln\left(1 - \frac{\mu_a\, k_a}{\beta_{\text{eff}}}\right), \tag{7}$$

which is finite and positive provided $\beta_{\text{eff}} > \mu_a\, k_a$ (else the activated steady state of cell j, β_{eff}/μ_a, lies below k_a and the relay fails altogether). Including the additional intracellular latency τ_{cell} from receptor activation to surface ligand presentation, the total time for one relay step is $T_{\text{step}} = \tau_{\text{cell}} + t_{\text{th}}$, and the resulting relay velocity scale is

$$\boxed{v_a = \frac{\ell_{\text{cell}}}{\tau_{\text{cell}} + t_{\text{th}}}} \tag{8}$$

in physical length units (with ℓ_{cell} the cell-to-cell distance), or $v_a = 1/(\tau_{\text{cell}} + t_{\text{th}})$ in cell-per-time units.

Limit behaviors. Three regimes follow directly from the form of t_{th}:

- *Well above threshold* ($\beta_{\text{eff}} \gg \mu_a\, k_a$): Taylor expansion of the logarithm gives $t_{\text{th}} \approx k_a/\beta_{\text{eff}}$, so $v_a \approx \ell_{\text{cell}}/(\tau_{\text{cell}} + k_a/\beta_{\text{eff}})$. The relay rate is dominated by production rate β_{eff} and sensitivity k_a; degradation enters only as a higher-order correction.
- *Near threshold* ($\beta_{\text{eff}} \to \mu_a\, k_a$ from above): the argument of the logarithm approaches zero, $t_{\text{th}} \to \infty$, and $v_a \to 0$. The relay slows and eventually fails.
- *Subthreshold* ($\beta_{\text{eff}} < \mu_a\, k_a$): the activated steady state β_{eff}/μ_a lies below k_a, and the relay cannot proceed at all.

This estimate captures the regime-dependent role of each circuit parameter, discussed in the next subsection.

Circuit parameters governing v_a

For fixed lattice geometry, contact topology, kernel weights, and front orientation, the threshold-crossing time t_{th}, and hence the relay velocity scale v_a, depend on five circuit parameters, all properties of the activation circuit rather than of the medium in which signaling occurs. The sensitivities below are computed at fixed s_j^*; in the full coupled dynamics, the same parameters also enter through the upstream activated-cell levels a_l^* and hence through s_j^* itself.

bioRxiv preprint doi: https://doi.org/10.64898/2026.05.24.727552; this version posted May 25, 2026. The copyright holder for this preprint (which was not certified by peer review) is the author/funder, who has granted bioRxiv a license to display the preprint in perpetuity. It is made available under a CC-BY 4.0 International license.

- **Activator production rate (β_a):** sets the effective production amplitude $\beta_{\text{eff}} = \beta_a\, F(s_j^*)$. Higher β_a raises β_{eff}, reduces t_{th}, and accelerates the relay. Well above threshold, $t_{\text{th}} \approx k_a/\beta_{\text{eff}} \propto 1/\beta_a$, so v_a grows monotonically with β_a and saturates at $v_a \to \ell_{\text{cell}}/\tau_{\text{cell}}$ as $\beta_a \to \infty$.
- **Cooperativity of activation (n_a):** sets the sharpness of the Hill response $F(s)$ around k_a, and shapes cell behavior on both sides of k_a, not only above it. At fixed input s_j^* (the quasi-static regime adopted here), only the value $F(s_j^*)$ enters $\beta_{\text{eff}} = \beta_a F(s_j^*)$, not the slope $\partial F/\partial s$, and the effect of n_a on the relay velocity depends on whether s_j^* sits above or below k_a:
  - $s_j^* > k_a$: $F(s_j^*)$ rises toward 1 faster as n_a increases, raising β_{eff} and accelerating the relay;
  - $s_j^* = k_a$: $F(s_j^*) = 1/2$ for all n_a, so cooperativity has no effect at the half-maximal input;
  - $s_j^* < k_a$: $F(s_j^*)$ falls toward 0 faster as n_a increases, lowering β_{eff} and slowing or preventing the relay.

  The local slope $\partial F/\partial s$ enters in a full dynamic front problem where $s_j(t)$ evolves during the threshold-crossing event, but does not contribute directly in the constant-input estimate.
- **Activation sensitivity (k_a):** the half-maximal input level of the Hill function (with kernel-geometry prefactors absorbed, as discussed in the Threshold-crossing estimate above). Higher k_a means lower sensitivity to a given juxtacrine input level, so cell j requires longer to accumulate enough activator to drive production in its next neighbor. This appears in the relay-time formula as $t_{\text{th}} = -(1/\mu_a)\ln(1 - \mu_a\, k_a/\beta_{\text{eff}})$, which increases monotonically with k_a. k_a also sets the propagation-failure boundary: when $\mu_a\, k_a$ exceeds β_{eff}, the activated steady state β_{eff}/μ_a falls below k_a and the relay cannot proceed.
- **Activator degradation rate (μ_a):** enters in two distinct ways. In the well-above-threshold regime, $t_{\text{th}} \approx k_a/\beta_{\text{eff}}$ and μ_a contributes only as a higher-order correction; the relay rate is essentially independent of degradation. Near threshold, higher μ_a lowers the activated steady state β_{eff}/μ_a toward k_a, increases t_{th} (through the logarithm), and slows the relay. Sufficiently high μ_a drives the activated steady state below k_a (equivalently, $\beta_{\text{eff}} < \mu_a\, k_a$), and the relay can no longer proceed. The role of μ_a is therefore best characterized as setting the regime in which the relay operates rather than as a leading-order multiplier of velocity.
- **Cell response time (τ_{cell}):** the latency from receiving juxtacrine signal to presenting sufficient surface ligand to a neighbor. This summarizes the intracellular kinetics of the relay step — receptor activation, downstream signaling, transcription, translation, and membrane trafficking of the new ligand. τ_{cell} enters the relay step time additively: $T_{\text{step}} = \tau_{\text{cell}} + t_{\text{th}}$. The effect of τ_{cell} on v_a therefore depends on the regime: when $\tau_{\text{cell}} \gg t_{\text{th}}$, the relay rate is set primarily by intracellular latency; when $t_{\text{th}} \gg \tau_{\text{cell}}$, by the threshold-crossing dynamics.

These five circuit parameters are genetically tunable, in principle: production rate through promoter strength and translation efficiency, cooperativity through receptor architecture, sensitivity through receptor affinity, degradation rate through degron tags or fusion partners, and cell response time through signaling pathway design. The realized tunability in any particular experimental system depends on cell type, trafficking, and other context-dependent factors. Stochastic integration and multiplicity of infection of lentiviral vectors primarily shape variation in production rate; the other parameters would need to be modified through changes in the protein sequences themselves.

bioRxiv preprint doi: https://doi.org/10.64898/2026.05.24.727552; this version posted May 25, 2026. The copyright holder for this preprint (which was not certified by peer review) is the author/funder, who has granted bioRxiv a license to display the preprint in perpetuity. It is made available under a CC-BY 4.0 International license.

Relationship to D_a and to circuit control

In PAPI, the activator's spatial behavior depends on both the molecular diffusion coefficient D_a (set by the molecular identity of the activator) and on circuit-encoded kinetic parameters (such as μ_a, which contributes to the diffusion length $\sqrt{D_a/\mu_a}$). D_a is a biophysical property of the activator molecule and is not accessible to genetic manipulation without changing the molecule itself — the central protein-engineering challenge for synthetic PAPI in mammalian cells.

In JAPI, D_a is absent by construction: the membrane-tethered activator does not diffuse. For fixed tissue geometry and fixed juxtacrine contact kernel K_{jl}, the kinetic parameters governing the activator's spatial behavior are all circuit-encoded (β_a, n_a, μ_a, k_a, τ_{cell}). This is a reduction in the total number of parameters available to control activator dynamics — JAPI offers fewer handles than PAPI — but the parameter that has been eliminated is the one that has historically been hardest to engineer. The remaining handles, in JAPI, are all genetically tunable rather than tied to molecular biophysics.

This architectural feature is what makes JAPI experimentally accessible in mammalian cells without requiring molecular engineering of differential diffusion coefficients.

Dynamic regime and scope

The propagation velocity v_a as derived above characterizes the relay rate in the inhibitor-suppressed limit $f(\cdot, i_j) \approx f(\cdot, 0)$, corresponding either to the early expansion phase of an activated domain before significant inhibitor accumulation, or to the transceiver configuration where the inhibitor branch is absent. As inhibitor accumulates around an expanding domain, $f(\cdot, i_j)$ is suppressed at the front by the local inhibitor level, the effective production β_{eff} drops, t_{th} grows, and the front eventually arrests. The arrest condition therefore depends critically on the inhibitor branch: on β_i, k_i, n_i, γ_i, and D_i, which together determine the spatial profile of inhibitor around an activated domain and the threshold at which β_{eff} falls below $\mu_a k_a$ and relay can no longer advance. The full arrest dynamics, including the determination of arrested domain size from the inhibitor profile, are addressed in Supplementary Note 4.

A full traveling-wave analysis yielding v_a in closed form would relax the quasi-static approximation for $s_j(t)$, treat the coupled dynamics of activator levels across the lattice self-consistently, incorporate lattice-discreteness effects (such as front pinning, anisotropy, and propagation failure under thresholding), and include corrections from inhibitor accumulation and explicit dependence on the inhibitor-side parameters. This is the subject of future theoretical work.

References

1. Kolmogorov, A. N., Petrovsky, I. G. & Piskunov, N. S. (1937). A study of the diffusion equation with increase in the amount of substance, and its application to a biological problem. *Bulletin of Moscow State University, Series A: Mathematics and Mechanics*, 1(6), 1–25.

2. Murray, J. D. (2003). *Mathematical Biology II: Spatial Models and Biomedical Applications* (3rd ed.). Springer-Verlag, New York.

bioRxiv preprint doi: https://doi.org/10.64898/2026.05.24.727552; this version posted May 25, 2026. The copyright holder for this preprint (which was not certified by peer review) is the author/funder, who has granted bioRxiv a license to display the preprint in perpetuity. It is made available under a CC-BY 4.0 International license.

Supplementary Note 3
Dimensionless Parameterization and Fixed Point Structure for Numerical Simulations

This note serves as a prerequisite for the numerical simulations. JAPI (juxtacrine-activator paracrine-inhibitor) and PAPI (paracrine-activator paracrine-inhibitor) are the two reaction-diffusion architectures compared throughout this paper; full definitions are given in Supplementary Note 1. Here we reduce the dimensional JAPI system to a minimal set of dimensionless groups, establish the fixed point structure in that parameter space, and define the parameter regimes explored in the numerical results. The PAPI nondimensionalization is given alongside for parallel reference. The linear stability analysis of Supplementary Note 1 is formulated in dimensional parameters and does not require this reduction; however, all numerical simulations are reported in the dimensionless units defined here.

1 Nondimensionalization

Dimensional JAPI system. In JAPI the activator is membrane-tethered, while the inhibitor is a diffusible field. Cells respond only to ligand presented on adjacent cells, not their own, so the autoactivation is non-cell-autonomous. The activator on cell j is induced only by activator-ligand presented on its *neighboring* cells through synNotch-ligand contact. Both species are produced by the same cell-autonomous response function f, evaluated on the signal received from neighbors and on the local inhibitor. With cells indexed by j on a lattice, the dimensional dynamics are:

$$\frac{da_j}{dt} = \beta_a\, f((Ka)_j,\, i_j) - \mu_a\, a_j, \tag{1}$$

$$\frac{\partial i}{\partial t} = D_i\, \frac{\partial^2 i}{\partial x^2} + \beta_i\, f((Ka)_j,\, i_j) - \gamma_i\, i, \tag{2}$$

where $i_j \equiv i(x_j, t)$, the Hill response function is (Alon, 2007)

$$f(a, i) = \frac{(a/k_a)^{n_a}}{1 + (a/k_a)^{n_a} + (i/k_i)^{n_i}}, \tag{3}$$

and the juxtacrine kernel sums activator over neighbors with no self-sensing:

$$(Ka)_j = \sum_l K_{jl}\, a_l, \qquad K_{jj} = 0. \tag{4}$$

Writing the kernel as a function of the integer cell separation $r = l - j$ (the coupling is translation-invariant on the lattice, so $K_{jl} = K(r)$), symmetric nearest-neighbor coupling on a 1D lattice gives $K(\pm 1) = 1/2$ and $K(r) = 0$ otherwise; each cell receives the average of its two neighbors. The support of K sets the spatial range over which the activator can act: the nearest-neighbor choice

bioRxiv preprint doi: https://doi.org/10.64898/2026.05.24.727552; this version posted May 25, 2026. The copyright holder for this preprint (which was not certified by peer review) is the author/funder, who has granted bioRxiv a license to display the preprint in perpetuity. It is made available under a CC-BY 4.0 International license.

fixes this activator interaction range to a single cell spacing by design, so it does not introduce a length scale competing with the inhibitor diffusion length. The total coupling weight is $\kappa \equiv \sum_l K_{jl} = 1$, so that homogeneous activator levels propagate unchanged through the kernel: $(Ka)_j = \kappa\, a_0 = a_0$ at any homogeneous state. Spatial position on the lattice is measured in units of cell-cell spacing, so the dimensionless lattice spacing is one by convention; the Fourier kernel eigenvalue $\hat{K}(q) = \cos(q)$ (Supplementary Note 1) follows from this convention, with q measured in radians per cell. Equations (1)–(2) write the activator on the discrete lattice and the inhibitor as a continuous diffusive field for analytical convenience and to match the formulation of the linear stability analysis; in numerical simulations both species are implemented on the same lattice with a discrete Laplacian for the inhibitor.

The dimensional system has seven parameters with units (β_a, β_i, μ_a, γ_i, D_i, k_a, k_i), plus two already-dimensionless Hill exponents (n_a and n_i).

Choice of scales. We rescale using the natural scales of the system (Murray, 2003):

$$\tilde{a} = \frac{a}{k_a}, \qquad \tilde{i} = \frac{i}{k_i}, \qquad \tilde{t} = \mu_a\, t, \qquad \tilde{x} = \frac{x}{\ell}, \quad \ell = \sqrt{\frac{D_i}{\mu_a}}. \tag{5}$$

The time scale $1/\mu_a$ is set by the activator's degradation rate. The two concentration scales k_a and k_i are the Hill thresholds for activation and inhibition. The characteristic length $\ell = \sqrt{D_i/\mu_a}$ is the distance an inhibitor molecule diffuses on the activator time scale; it is *not* the same as the dimensional inhibitor diffusion length $\lambda_i = \sqrt{D_i/\gamma_i}$, which represents the distance an inhibitor diffuses in its own lifetime. The latter appears in dimensionless units as $1/\sqrt{\gamma}$ (Section 3).

Substitution: activator equation. Substituting (5) into (1) and dividing through by $k_a\mu_a$:

$$\frac{da_j}{dt} = \underbrace{\frac{\beta_a}{k_a\,\mu_a}}_{=r_a} f((Ka)_j, i_j) - a_j, \tag{6}$$

where tildes have been dropped: a and i now denote the dimensionless variables of (5), with the Hill thresholds k_a and k_i absorbed into their definition (equivalently, $k_a = k_i = 1$ in these units). The response function (3) accordingly reduces to

$$f(a, i) = \frac{a^{n_a}}{1 + a^{n_a} + i^{n_i}}, \tag{7}$$

which we use throughout the remainder of the note. The kernel weights K_{jl} are dimensionless lattice quantities set by geometry and so are unchanged by the rescaling.

Substitution: inhibitor equation. Substituting (5) into (2), the diffusion coefficient picks up a factor $D_i/(\mu_a\,\ell^2)$:

$$\frac{\partial i}{\partial t} = \underbrace{\frac{D_i}{\mu_a\,\ell^2}}_{=1 \text{ by choice of } \ell} \frac{\partial^2 i}{\partial x^2} + \underbrace{\frac{\beta_i}{k_i\,\mu_a}}_{=r_i} f((Ka)_j, i_j) - \underbrace{\frac{\gamma_i}{\mu_a}}_{=\gamma} i. \tag{8}$$

The choice $\ell = \sqrt{D_i/\mu_a}$ makes $D_i/(\mu_a\ell^2) = 1$ exactly. This is what is meant by "D_i has been absorbed into the length scale": D_i does not appear as a free parameter in the dimensionless equations because it has been used up defining ℓ; the entire inhibitor diffusion strength now lives in the choice of length unit.

bioRxiv preprint doi: https://doi.org/10.64898/2026.05.24.727552; this version posted May 25, 2026. The copyright holder for this preprint (which was not certified by peer review) is the author/funder, who has granted bioRxiv a license to display the preprint in perpetuity. It is made available under a CC-BY 4.0 International license.

Dimensionless JAPI system. Collecting:

$$\frac{da_j}{dt} = r_a\, f((Ka)_j, i_j) - a_j, \tag{9}$$

$$\frac{\partial i}{\partial t} = \frac{\partial^2 i}{\partial x^2} + r_i\, f((Ka)_j, i_j) - \gamma\, i, \tag{10}$$

with five dimensionless groups:

$$r_a = \frac{\beta_a}{k_a\, \mu_a}, \quad r_i = \frac{\beta_i}{k_i\, \mu_a}, \quad \gamma = \frac{\gamma_i}{\mu_a}, \quad n_a, \quad n_i. \tag{11}$$

r_a is the dimensionless activator drive (the production-to-degradation ratio); r_i is the analogous quantity for the inhibitor; γ is the ratio of inhibitor to activator degradation rates, governing how quickly the inhibitor decays relative to the activator; n_a and n_i govern the steepness of the activation and inhibition responses. The activator diffusion coefficient D_a does not appear: it has been eliminated by the JAPI architecture itself, where the activator is membrane-bound rather than diffusible (Supplementary Note 1).

Comparison with PAPI. In PAPI, the activator is also a diffusible field, with diffusion coefficient D_a. The dimensional inventory adds D_a, and the activator equation gains a Laplacian term. Applying the same rescaling (which uses $\ell = \sqrt{D_i/\mu_a}$ as the unit of length in both architectures) yields the dimensionless PAPI system:

$$\frac{\partial a}{\partial t} = \frac{1}{D}\frac{\partial^2 a}{\partial x^2} + r_a\, f(a,i) - a, \tag{12}$$

$$\frac{\partial i}{\partial t} = \frac{\partial^2 i}{\partial x^2} + r_i\, f(a,i) - \gamma\, i, \tag{13}$$

with one additional dimensionless group:

$$D = \frac{D_i}{D_a}, \tag{14}$$

the inhibitor-to-activator diffusion ratio. We adopt the convention $D = D_i/D_a$ (rather than its reciprocal) so that the Turing-favorable regime corresponds to $D > 1$, consistent with classical reaction-diffusion literature. PAPI therefore has six dimensionless groups: r_a, r_i, γ, D, n_a, n_i. The same rescaling absorbs D_i into the length scale in both architectures; the parameter D_a that distinguishes PAPI cannot be absorbed into the same length scale and survives as the residual ratio $D = D_i/D_a$.

2 Steady State Analysis in Dimensionless Form

Setting spatial gradients to zero, the dimensionless steady states satisfy:

$$r_a\, f(a_0, i_0) = a_0 \tag{15}$$

$$r_i\, f(a_0, i_0) = \gamma\, i_0 \tag{16}$$

Trivial fixed point. $(a_0, i_0) = (0,0)$ always satisfies (15), (16). For $n_a > 1$, the activator production $f(a,i) \sim a^{n_a}$ vanishes faster than linearly near the origin, so the linearized dynamics carry no production term and the trivial fixed point is linearly stable; this state therefore does not support Turing instability in the regimes considered here (Supplementary Note 1). The $n_a = 1$ case is parameter-dependent and is not considered.

bioRxiv preprint doi: https://doi.org/10.64898/2026.05.24.727552; this version posted May 25, 2026. The copyright holder for this preprint (which was not certified by peer review) is the author/funder, who has granted bioRxiv a license to display the preprint in perpetuity. It is made available under a CC-BY 4.0 International license.

Reduction to a scalar root-finding problem. Dividing (15) by (16) gives

$$i_0 = \frac{r_i}{\gamma\, r_a}\, a_0, \tag{17}$$

so any nontrivial fixed point lies on this line in the (a_0, i_0) plane. The two steady-state equations therefore reduce to a scalar root-finding problem along (17); the dynamics themselves remain two-dimensional.

Nontrivial fixed points. Substituting (17) into (15) and dividing through by the overall factor of a (which separates the trivial root $a_0 = 0$ already accounted for above), the nontrivial fixed points are the positive roots of

$$g(a) \equiv 1 + a^{n_a} + \left(\frac{r_i}{\gamma\, r_a}\right)^{n_i} a^{n_i} - r_a\, a^{n_a - 1} = 0. \tag{18}$$

Note that $g(0) = 1 \neq 0$: the trivial root lives in the full equation $a \cdot g(a) = 0$, not in g itself. For integer Hill exponents n_a, n_i, Descartes' rule of signs admits 0, 1 (a double root), or 2 positive real roots, corresponding to the monostable, saddle-node, and bistable cases respectively; for non-integer Hill exponents the same count holds by a generalized argument on the sign changes of $g(a)$.

Saddle-node boundary. In the $(r_a, r_i/\gamma)$ parameter plane (at fixed n_a, n_i), the boundary between monostable and bistable regimes is the set on which (18) admits a positive double root, i.e. $g(a) = 0$ and $g'(a) = 0$ hold simultaneously. We solve this pair numerically for each (n_a, n_i) to map the bistable region used in the simulations.

Activator-only limit. In the limit $r_i \to 0$, (18) reduces to $1 + a^{n_a} - r_a\, a^{n_a - 1} = 0$, and the saddle-node tangency between the production curve $r_a f(a, 0) = r_a a^{n_a}/(1 + a^{n_a})$ and the degradation line a (Strogatz, 1994) gives the closed-form thresholds:

$$a_c = (n_a - 1)^{1/n_a} \tag{19}$$

$$r_a^c = \frac{n_a\,(n_a - 1)^{1/n_a}}{n_a - 1} \tag{20}$$

r_a^c is therefore the saddle-node boundary in the activator-only slice $r_i = 0$, and serves as a lower-bound calibration for the full inhibited boundary: for $r_i > 0$, the saddle-node boundary lies above r_a^c in the $(r_a, r_i/\gamma)$ plane. The critical value r_a^c decreases monotonically toward 1 as n_a increases, so higher cooperativity makes bistability easier to achieve in this limit.

Fixed-point structure in the bistable region. In the bistable region of the inhibited $(r_a, r_i/\gamma)$ plane, the system has three fixed points along the line (17): the stable off state at $(0, 0)$, an unstable intermediate fixed point at the smaller positive root of g, and a stable activated state at the larger positive root, with $a_0 \to r_a$ and $i_0 \to r_i/\gamma$ for r_a well above the boundary. Stability classifications follow from the standard 2×2 Jacobian analysis of (15)–(16) (Strogatz, 1994) and are not reproduced here.

Effect of inhibition. Increasing r_i/γ shifts the saddle-node boundary toward larger r_a, shrinking the bistable region. Mechanically, the inhibitor suppresses the production curve at any nontrivial fixed point relative to the activator-only case (Gierer & Meinhardt, 1972), raising the value of the unstable intermediate fixed point in a and shifting the separatrix between the two basins of attraction toward the activated state.

bioRxiv preprint doi: https://doi.org/10.64898/2026.05.24.727552; this version posted May 25, 2026. The copyright holder for this preprint (which was not certified by peer review) is the author/funder, who has granted bioRxiv a license to display the preprint in perpetuity. It is made available under a CC-BY 4.0 International license.

3 Parameter Space for Numerical Simulations

The five dimensionless groups (11) define the parameter space explored numerically; for PAPI, the additional group $D = D_i/D_a$ is included, fixed at $D = 10$ across the scan (a standard Turing-favorable regime in classical reaction-diffusion literature). Simulations span both the monostable and bistable regimes, separated by the saddle-node boundary in the $(r_a, r_i/\gamma)$ plane (Section 2), which reduces to $r_a = r_a^c$ in the activator-only limit $r_i \to 0$. The scan is systematic across the full parameter space (cf. Marcon et al., 2016); visualizations of patterning outcomes are necessarily lower-dimensional projections, with two or three of the groups held fixed to render a 2D plane or 3D volume. The choice of which dimensions to display is dictated by visualization rather than by physics, and the held-fixed values are reported alongside the corresponding figure. Full numerical setup (lattice dimensions, boundary conditions, initial-condition protocol, noise amplitude, time horizon, solver and tolerances, and pattern-classification criteria) is specified in Methods. JAPI and PAPI share the same homogeneous fixed-point structure (Section 2) but differ in their spatial dispersion: in JAPI the activator coupling enters multiplicatively as $r_a f_a \hat{K}(q)$, while in PAPI it enters additively as $-(1/D)\,\Lambda(q)$. At small q the JAPI cosine kernel can be locally matched to an effective activator diffusion coefficient ($\hat{K}(q) = 1 - q^2/2 + O(q^4)$, equivalent to a continuum diffusion $D_a^{\text{eff}} = r_a f_a/2$ at long wavelengths; Supplementary Note 1), but the full dispersion relations and nonlinear couplings are not equivalent at any single value of D, and the two systems are scanned independently.

Two distinct lengths appear in this formulation: the rescaling length $\ell = \sqrt{D_i/\mu_a}$ used to define dimensionless space (Section 1), and the dimensional inhibitor diffusion length $\lambda_i = \sqrt{D_i/\gamma_i}$, the distance an inhibitor molecule diffuses in its own lifetime. In dimensionless units, $\lambda_i/\ell = 1/\sqrt{\gamma}$, which sets the natural spatial scale of domain size in the irregular patterning regime (Supplementary Note 2). All spatial outputs (domain widths, inter-feature spacing, pattern wavelengths) are reported in dimensionless units, i.e. in multiples of ℓ.

References

1. Gierer, A. & Meinhardt, H. (1972). A theory of biological pattern formation. *Kybernetik*, 12(1), 30–39.

2. Strogatz, S. H. (1994). *Nonlinear Dynamics and Chaos: With Applications to Physics, Biology, Chemistry, and Engineering*. Addison-Wesley, Reading, MA.

3. Murray, J. D. (2003). *Mathematical Biology II: Spatial Models and Biomedical Applications* (3rd ed.). Springer-Verlag, New York.

4. Alon, U. (2007). *An Introduction to Systems Biology: Design Principles of Biological Circuits*. Chapman & Hall/CRC, Boca Raton, FL.

5. Marcon, L., Diego, X., Sharpe, J. & Müller, P. (2016). High-throughput mathematical analysis identifies Turing networks for patterning with equally diffusing signals. *eLife*, 5, e14022.

bioRxiv preprint doi: https://doi.org/10.64898/2026.05.24.727552; this version posted May 25, 2026. The copyright holder for this preprint (which was not certified by peer review) is the author/funder, who has granted bioRxiv a license to display the preprint in perpetuity. It is made available under a CC-BY 4.0 International license.

Supplementary Note 4
Domain Arrest in the Irregular Patterning Regime of Reaction-Diffusion Systems

This note addresses a question raised by the experimental observation that JAPI circuits implemented in mammalian cells produce robust patterns despite operating in a parameter regime where linear stability analysis classifies the homogeneous activated state as stable. The same regime is accessible numerically in both JAPI and PAPI architectures and gives rise to non-periodic, irregular patterns formed of finite-sized activated domains distributed in space (see main text Fig. 2D for example). This note provides a heuristic backbone of an analytical mechanism by which this occurs.

The analysis is general to reaction-diffusion systems with a diffusing inhibitor and applies to both JAPI and PAPI architectures: the inhibitor-field calculation requires only β_i, D_i, γ_i, and the activation threshold I_c, not the form of the activator spatial coupling. Architecture-specific content enters the result in two places: (i) through the activator-side parameters that set I_c (the inhibitor concentration that prevents activation at the front, which depends on the receiver cell's production rate, degradation rate, and Hill response), and (ii) through the activator front velocity v, which determines whether the quasi-static approximation underlying the closed-form result is valid. Within the quasi-static regime (defined below), the closed-form arrested domain size depends on $(\lambda_i, \beta_i, I_c)$ only; v does not appear at leading order. Throughout this note, we treat the activator front velocity as an abstract quantity v representing the rate at which the activated domain expands. In the JAPI architecture, v reaches its maximum value, v_a (Supplementary Note 2), in the inhibitor-free limit; during pattern formation v is bounded above by v_a and decreases as inhibitor accumulates around the expanding domain.

The analysis is restricted to the irregular regime, where individual domains form and stabilize in isolation. The irregular regime arises from both monostable and bistable parameter conditions in JAPI/PAPI architectures; we comment on the implications for stability at the end of the existence-condition section. It does not apply to the Turing regime, where domain boundaries are set by the dispersion relation rather than by self-consistent inhibitor accumulation.

Setup: an isolated activated domain in steady state

In the irregular regime, patterns form by nucleation: local fluctuations cross a threshold, an activated domain appears, expands by relay or diffusion of the activator, and produces inhibitor that accumulates around it. We analyze a single such domain treated in isolation, assuming it has reached a steady state in which:

- The domain has half-width R and is bounded by an activation front
- Inside the domain, the activator is at its activated steady state, so all cells within the domain produce inhibitor at the maximum rate β_i

bioRxiv preprint doi: https://doi.org/10.64898/2026.05.24.727552; this version posted May 25, 2026. The copyright holder for this preprint (which was not certified by peer review) is the author/funder, who has granted bioRxiv a license to display the preprint in perpetuity. It is made available under a CC-BY 4.0 International license.

- Outside the domain, no cells are activated and no inhibitor is produced locally
- The inhibitor field is treated as quasi-static, instantly tracking the slowly evolving domain geometry

We treat the inhibitor field as quasi-static, instantly tracking the slowly evolving domain geometry. This corresponds to the slow-front limit $v \ll \sqrt{D_i \gamma_i}$, where the inhibitor relaxation time $1/\gamma_i$ is much shorter than the timescale on which the activation front advances by one diffusion length. This is a simplifying limit adopted to enable the closed-form solution presented below; the validity of this assumption for the experimental system, the corrections at finite v, and the breakdown of the quasi-static framework at high v are addressed in the final section of this note.

We additionally treat the activation front as a locally planar one-dimensional boundary. This approximation is exact for stripe-like domains and accurate for compact two- or three-dimensional domains when the boundary curvature radius is large compared to the inhibitor diffusion length λ_i. For compact two-dimensional domains with $R \sim \lambda_i$, curvature corrections modify the boundary inhibitor concentration; the leading-order result of the planar calculation captures the qualitative dependence on β_i, λ_i, and I_c, while quantitative agreement requires the full Green's-function solution. We adopt the planar approximation for transparency and analytical tractability; numerical simulations in the main text (Fig. 2L-M) do not rely on it.

In one dimension, treating the domain interior as a region of constant inhibitor production and the exterior as a region with no production, the inhibitor concentration $I(x)$ satisfies the linear equation:

$$D_i \frac{d^2 I}{dx^2} - \gamma_i I + \beta_i \, \mathbf{1}_{|x|<R} = 0 \tag{1}$$

where $\mathbf{1}_{|x|<R}$ is the indicator function of the domain. The natural length scale is the inhibitor diffusion length:

$$\lambda_i = \sqrt{D_i/\gamma_i} \tag{2}$$

the characteristic distance over which the inhibitory halo around the domain decays. The parameters used throughout this analysis are summarized in Table 1.

R	domain half-width
$I(x)$	inhibitor concentration
β_i	inhibitor production rate inside activated cells
D_i	inhibitor diffusion coefficient
γ_i	inhibitor degradation rate
$\lambda_i = \sqrt{D_i/\gamma_i}$	inhibitor diffusion length
I_c	inhibitor concentration that blocks activation at the domain boundary
v_a	inhibitor-free activator front velocity
$\rho = 2\gamma_i I_c/\beta_i$	dimensionless arrest threshold (introduced below)

Table 1: Parameters used in the arrest analysis.

Solving for the boundary inhibitor concentration

Equation (1) is solvable exactly. By symmetry the solution is even in x. Inside the domain, the general solution combines a particular solution $I_p = \beta_i/\gamma_i$ with the even homogeneous mode

bioRxiv preprint doi: https://doi.org/10.64898/2026.05.24.727552; this version posted May 25, 2026. The copyright holder for this preprint (which was not certified by peer review) is the author/funder, who has granted bioRxiv a license to display the preprint in perpetuity. It is made available under a CC-BY 4.0 International license.

$\cosh(x/\lambda_i)$. Outside the domain, the solution decays exponentially as $e^{-(|x|-R)/\lambda_i}$ to satisfy $I(x) \to 0$ at infinity. Matching I and I' at $x = R$ and solving the resulting linear system gives:

$$I(R) = \frac{\beta_i}{2\gamma_i}\left(1 - e^{-2R/\lambda_i}\right) \tag{3}$$

The boundary value has two clean limits. For small domains ($R \ll \lambda_i$), Taylor expansion gives $I(R) \approx (\beta_i/\gamma_i) \cdot (R/\lambda_i)$, so the boundary inhibitor grows linearly with domain size, since the entire domain contributes to the inhibitor field at the edge. For large domains ($R \gg \lambda_i$), the exponential vanishes and $I(R) \to \beta_i/(2\gamma_i)$, so the boundary value saturates, because cells more than a few λ_i away from the boundary do not contribute to it. The factor of one-half reflects the symmetric leakage of inhibitor outward from the boundary.

The arrest condition

The expanding front stalls when the inhibitor concentration at the boundary reaches a critical threshold I_c sufficient to prevent further activation: $I(R) = I_c$. I_c is itself a derived quantity that depends on the activator-side parameters governing the receiver cell's response (production rate, degradation rate, Hill parameters of activation): the inhibitor level at which the receiver fails to activate is set by the balance between inhibition and the activator signal arriving from the neighbor. Through this dependence, activator-side parameters enter the arrest condition (Eq. 6) by setting I_c, separately from the velocity channel discussed in the regime structure section below. This note takes I_c as an empirical input to the arrest analysis; deriving I_c from circuit parameters is part of the future theoretical work. Substituting (3) gives the arrest condition:

$$\beta_i\left(1 - e^{-2R/\lambda_i}\right) = 2\gamma_i I_c \tag{4}$$

which can be written compactly as $\beta_i h(R/\lambda_i) = \gamma_i I_c$ with the dimensionless geometric factor $h(x) = (1 - e^{-2x})/2$. We note that nucleation, the formation of the initial activated domain that this analysis assumes as its starting point, requires perturbations above a finite threshold, and is documented numerically in main text Fig. S7C-D rather than analytically here.

Existence condition for arrest

Equation (4) has an immediate consequence. Since $1 - e^{-2R/\lambda_i} < 1$ for any finite R, arrest is only possible when:

$$I_c < \frac{\beta_i}{2\gamma_i} \tag{5}$$

If the activation threshold I_c exceeds half the maximum interior inhibitor concentration β_i/γ_i, the boundary inhibitor can never reach I_c regardless of how large the domain grows. The front never stalls and the system proceeds to uniform activation. This existence condition therefore defines a quantitative boundary in parameter space between conditions where isolated domains arrest at finite size and conditions where activation propagates indefinitely until the system reaches uniform activation.

We note that equation (6), derived in the next section, identifies the half-width at which the arrest condition is satisfied; stability of the arrested state is addressed there.

bioRxiv preprint doi: https://doi.org/10.64898/2026.05.24.727552; this version posted May 25, 2026. The copyright holder for this preprint (which was not certified by peer review) is the author/funder, who has granted bioRxiv a license to display the preprint in perpetuity. It is made available under a CC-BY 4.0 International license.

Domain size when arrest occurs

When (5) is satisfied, solving (4) explicitly for R:

$$R = \frac{\lambda_i}{2} \ln\left(\frac{\beta_i}{\beta_i - 2\gamma_i I_c}\right) \tag{6}$$

In dimensionless form, defining $\rho = 2\gamma_i I_c/\beta_i$ as the dimensionless arrest threshold (satisfying $0 < \rho < 1$ when the existence condition holds), Eq. (6) becomes

$$\frac{R}{\lambda_i} = \frac{1}{2} \ln\left(\frac{1}{1-\rho}\right). \tag{7}$$

This separates the length scale λ_i from the dimensionless arrest threshold ρ, and makes transparent that the existence-condition divergence ($R \to \infty$ as $\rho \to 1^-$) corresponds to approaching the boundary $I_c \to \beta_i/(2\gamma_i)$ from below.

The qualitative dependencies on independent parameters follow directly: increasing D_i at fixed β_i, γ_i, I_c increases λ_i while leaving ρ unchanged, so R increases proportionally. Increasing β_i at fixed D_i, γ_i, I_c decreases ρ, so R decreases. Increasing I_c at fixed β_i, γ_i, D_i increases ρ, so R increases. The effect of changing γ_i in isolation is ambiguous because γ_i enters both λ_i and ρ; the dimensionless form clarifies that the net effect depends on which other quantities are held fixed.

Stability of the arrested state

Equation (6) identifies the half-width at which the arrest condition $I(R) = I_c$ is satisfied; it does not by itself establish dynamical stability of the arrested state. Local stability requires a boundary-motion law (e.g., $dR/dt = V(I_c - I(R))$ with $V(0) = 0$ and $V'(0) > 0$) and analysis of small perturbations to R. Because $dI/dR > 0$ in the planar quasi-static calculation, the geometric condition for dynamical stability is satisfied: a small increase in R raises the boundary inhibitor above I_c, suppressing further expansion; a small decrease lowers it below I_c, releasing the front. A formal stability analysis with an explicit front-motion law is the subject of future theoretical work.

Empirically, arrested domains persist in numerical simulations across the parameter ranges examined here (main text Fig. 2 and Fig. S7), consistent with dynamical stability in both the bistable and monostable regimes accessible to the system. In the bistable case, the arrested configuration sits between two homogeneous steady states; in the monostable case, persistence of arrested domains arises despite the absence of an alternative stable homogeneous state, distinguishing the irregular regime from a simple bistable picture.

Activator front velocity and the regime structure of arrest

The closed-form arrest condition derived above (Eq. 6) makes no reference to the activator front velocity v: R is determined entirely by inhibitor parameters and the activation threshold. In the quasi-static limit, the arrested domain size follows from the geometric self-consistency condition $I(R) = I_c$, which involves only the inhibitor field; the role of v is to set how quickly arrest is reached, not what size it stalls at.

This v-independence holds only in the quasi-static regime, $v \ll \sqrt{D_i\gamma_i}$. Because v varies in time during expansion (reaching its maximum v_a in the inhibitor-free limit at the start of the front's advance, and approaching zero near arrest), we use v_a as the relevant v in regime comparisons: it is the maximum value v takes during expansion, and serves as a conservative upper bound.

bioRxiv preprint doi: https://doi.org/10.64898/2026.05.24.727552; this version posted May 25, 2026. The copyright holder for this preprint (which was not certified by peer review) is the author/funder, who has granted bioRxiv a license to display the preprint in perpetuity. It is made available under a CC-BY 4.0 International license.

If $v_a/\sqrt{D_i\gamma_i}$ places the system in regime (a), the actual $v(t)/\sqrt{D_i\gamma_i}$ does so at all times during expansion. Three regimes can be distinguished:

- **(a) Quasi-static, $v_a \ll \sqrt{D_i\gamma_i}$:** Equation (6) applies at leading order; R does not depend on v. A heuristic order-of-magnitude estimate for the subleading correction is $\Delta R \sim v_a/\gamma_i$, valid in the bulk of regime (a) where dI/dR remains of order $\beta_i/(\gamma_i\lambda_i)$. This estimate breaks down near the existence boundary ($\rho \to 1^-$), where dI/dR vanishes and small finite-velocity reductions in boundary inhibitor can produce disproportionately large corrections to R. A rigorous moving-front derivation including the velocity-dependent boundary inhibitor concentration is the subject of future theoretical work.

- **(b) Lagging, $v_a \sim \sqrt{D_i\gamma_i}$:** The boundary inhibitor field is substantially below its quasi-static value at any given R. R remains finite but is larger than equation (6) predicts and depends on v in a nontrivial nonlinear way.

- **(c) Runaway, $v_a \gtrsim v_{\mathbf{crit}} \sim \sqrt{D_i\gamma_i}$:** The boundary inhibitor saturates at a value below I_c, the arrest condition is never satisfied, and the domain expands without bound until external constraints (tissue size, depletion of resources) intervene.

The transition from (b) to (c) is set by a critical velocity v_{crit} at which the dynamically saturated boundary inhibitor falls below I_c. Determining v_{crit} explicitly, and the full functional form $R(v)$ in regime (b), requires a traveling-wave analysis and is left to future theoretical work.

In regime (a) the dependence of R on inhibitor parameters takes a simple form: R increases monotonically with λ_i and decreases monotonically with β_i. The dependence on v is regime-specific: R is approximately v-independent at leading order in regime (a), depends nontrivially on v in regime (b), and is undefined in regime (c).

Empirical placement of JAPI. For the JAPI experimental system, the propagation velocity in L929 fibroblast transceivers (a configuration that lacks the inhibitor branch and therefore reports v_a directly) is $v_a \sim 0.13$ mm/day (Santorelli et al., 2024), equivalent to $\sim 1.5 \times 10^{-3}$ μm/s. Using inhibitor parameters consistent with engineered secreted morphogens (diffusion $D_i \sim 10$ μm^2/s, half-life $t_{1/2} \sim$ 2–6 hours, comparable to measured values for canonical Lefty in zebrafish; Müller et al., 2012), we estimate the inhibitor degradation rate as $\gamma_i = \ln(2)/t_{1/2} \approx 3.2 \times 10^{-5}$ to 9.6×10^{-5} s^{-1}, giving $\sqrt{D_i\gamma_i} \approx 1.8\times10^{-2}$ to 3.1×10^{-2} μm/s. The dimensionless ratio $v_a/\sqrt{D_i\gamma_i}$ therefore falls in the range $\sim$ 0.05–0.08, placing the system in regime (a): equation (6) applies as the leading-order description and arrest occurs robustly. Finite-velocity corrections are bounded in the bulk of regime (a): the heuristic lag scale v_a/γ_i evaluates to $\sim$ 16–47 μm, a fraction of the domain half-width $R \sim$ 50–100 μm (the half-width corresponding to arrested domain diameters of $\sim$ 100–200 μm), suggesting bulk corrections to R of order $\sim$ 30–50% when evaluated with the conservative upper-bound velocity v_a. The actual correction is likely smaller still, since the front velocity near arrest is well below v_a; corrections near the existence boundary $\rho \to 1$ require the moving-front analysis deferred to future theoretical work. These are order-of-magnitude estimates; precise placement would require direct measurement of the diffusion coefficient and degradation rate of the specific inhibitor used in the JAPI implementation. The architectural distinction between JAPI and PAPI enters here through both I_c (set by activator-side parameters of each architecture) and v_a: in PAPI, v_a scales as the square root of the activator diffusion coefficient times the activator's linear growth rate (the Fisher-KPP front scaling) and therefore depends on D_a, while in JAPI, D_a is absent and v_a is set by the parameters of Supplementary Note 2.

bioRxiv preprint doi: https://doi.org/10.64898/2026.05.24.727552; this version posted May 25, 2026. The copyright holder for this preprint (which was not certified by peer review) is the author/funder, who has granted bioRxiv a license to display the preprint in perpetuity. It is made available under a CC-BY 4.0 International license.

Biological summary

The arrest mechanism analyzed here gives a simple picture of what controls domain size in the irregular regime of reaction-diffusion systems:

- **Stronger inhibitor production** (higher β_i) $\longrightarrow$ boundary inhibitor reaches I_c at smaller R $\longrightarrow$ smaller domains.
- **Wider inhibitor reach** (higher λ_i at fixed dimensionless threshold $\rho = 2\gamma_i I_c/\beta_i$) $\longrightarrow$ inhibitor halo extends further $\longrightarrow$ larger domains. Increasing D_i at fixed β_i, γ_i, I_c increases R proportionally to λ_i; the effect of changing γ_i in isolation depends on which other quantities are held fixed, since γ_i enters both λ_i and the dimensionless threshold ρ.
- **Front velocity** affects domain size in a regime-specific way: R is approximately independent of v in the quasi-static regime ($v_a \ll \sqrt{D_i\gamma_i}$), depends nontrivially on v when v_a is comparable to $\sqrt{D_i\gamma_i}$, and arrest fails entirely when v_a exceeds a critical value. The relevant comparison uses v_a, the inhibitor-free upper bound on the front velocity; the actual front velocity during expansion is bounded above by v_a and approaches zero near arrest.
- **Arrest is only possible when $I_c < \beta_i/(2\gamma_i)$.** When this condition fails, the front cannot be stalled and the system proceeds to uniform activation.

This analysis applies to both JAPI and PAPI architectures, with architecture entering through two channels: I_c (set by the activator-side parameters governing the receiver cell's response) and v_a (set by how the activator's spatial coupling generates a front velocity). The inhibitor-field calculation itself is architecture-independent. Within regime (a), the closed-form result for R (Eq. 6) depends on $(\lambda_i, \beta_i, I_c)$; v_a does not appear at leading order, but determines regime placement. A formal derivation of v_a in terms of activator-side circuit parameters, together with the activator-side derivation of I_c and corrections from inhibitor coupling, is the subject of future theoretical work. In JAPI, v_a is set by a smaller number of parameters since D_a is absent, making domain size control more parsimonious through this channel.

References

1. Müller, P., Rogers, K. W., Jordan, B. M., Lee, J. S., Robson, D., Ramanathan, S. & Schier, A. F. (2012). Differential diffusivity of Nodal and Lefty underlies a reaction-diffusion patterning system. *Science*, 336(6082), 721–724.

2. Santorelli, M., Bhamidipati, P. S., Courte, J., Swedlund, B., Jain, N., Poon, K., Schildknecht, D., Kavanagh, A., MacKrell, V. A., Sondkar, T., Malaguti, M., Quadrato, G., Lowell, S., Thomson, M. & Morsut, L. (2024). Control of spatio-temporal patterning via cell growth in a multicellular synthetic gene circuit. *Nature Communications*, 15, 9867.
